# Bacterial Quorum-Sensing Regulation Induces Morphological Change in a Key Host Tissue during the Euprymna scolopes-Vibrio fischeri Symbiosis

**DOI:** 10.1128/mBio.02402-21

**Published:** 2021-09-28

**Authors:** T. Essock-Burns, B. D. Bennett, D. Arencibia, S. Moriano-Gutierrez, M. Medeiros, M. J. McFall-Ngai, E. G. Ruby

**Affiliations:** a Pacific Biosciences Research Center, University of Hawaiʻi at Mānoa, Honolulu, Hawaiʻi, USA; University of Connecticut

**Keywords:** morphogenesis, luminescence, actin polymerization, *Aliivibrio*

## Abstract

Microbes colonize the apical surfaces of polarized epithelia in nearly all animal taxa. In one example, the luminous bacterium Vibrio fischeri enters, grows to a dense population within, and persists for months inside, the light-emitting organ of the squid Euprymna scolopes. Crucial to the symbiont’s success after entry is the ability to trigger the constriction of a host tissue region (the “bottleneck”) at the entrance to the colonization site. Bottleneck constriction begins at about the same time as bioluminescence, which is induced in V. fischeri through an autoinduction process called quorum sensing. Here, we asked the following questions: (i) Are the quorum signals that induce symbiont bioluminescence also involved in triggering the constriction? (ii) Does improper signaling of constriction affect the normal maintenance of the symbiont population? We manipulated the presence of three factors, the two V. fischeri quorum signal synthases, AinS and LuxI, the transcriptional regulator LuxR, and light emission itself, and found that the major factor triggering and maintaining bottleneck constriction is an as yet unknown effector(s) regulated by LuxIR. Treating the animal with chemical inhibitors of actin polymerization reopened the bottlenecks, recapitulating the host’s response to quorum-sensing defective symbionts, as well as suggesting that actin polymerization is the primary mechanism underlying constriction. Finally, we found that these host responses to the presence of symbionts changed as a function of tissue maturation. Taken together, this work broadens our concept of how quorum sensing can regulate host development, thereby allowing bacteria to maintain long-term tissue associations.

## INTRODUCTION

Perhaps the most widespread type of animal-microbe symbiosis is the association of extracellular bacteria along the apical surfaces of epithelial tissue (1). In such symbioses, the biochemical conversation between the microbes and their host can be strongly dependent on an underlying level of bacterium-bacterium signaling. For example, the well-described process of quorum sensing (QS) by Gram-negative species not only regulates density-dependent group behaviors of bacterial populations (2) but can also change how and when those populations interact with host tissues, either in pathogenesis or in mutualism (3–7).

The first recognized bacterial QS signal (also called an autoinducer), 3-oxo-hexanoyl homoserine lactone (3O-C6), was discovered in *Vibrio* (*Aliivibrio*) *fischeri*, a marine species that induces bioluminescence in environments where it can achieve a high cell density (8). Subsequent study of V. fischeri revealed the presence of a second QS molecule, octanoyl homoserine lactone (C8), which controls the induction of light emission in a signal transduction cascade upstream of the 3O-C6 inducer (9). The two signals are continuously released by the bacterium, and their accumulation works sequentially (10) to regulate a suite of genes in the symbiont population (11). The V. fischeri QS network shares homology with those pathways of other *Proteobacteria*, many of which form either pathogenic or beneficial associations with animals or plants (12). For example, V. fischeri is the only bacterium that can colonize the confined crypt spaces inside the light-emitting organ of the bobtail squid Euprymna scolopes (13). Within these spaces, the 3O-C6 accumulates around the dense population of symbionts, activating their *luxICDABEG* operon and resulting in a bioluminescence used by the host in its nocturnal behavior (14, 15). Bacteria with mutations in the genes encoding the synthases of either C8 (*ainS*) or 3O-C6 (*luxI*) (16) can still initiate colonization; however, after 24 h, the *luxI* mutation results in a symbiotic persistence defect (4, 10).

Studies of the squid-vibrio system have suggested that the 3O-C6 signal released by the symbionts may directly influence the expression of a few host genes; however, the major effects of 3O-C6 result from binding to its cognate bacterial transcriptional regulator LuxR, which then induces symbiont activities that indirectly drive host transcriptomic responses (18) and morphological development (4). While bacterial luminescence is one such driving activity (19), the impact of other QS-induced symbiont functions on the host’s ability to develop and maintain a productive symbiosis is not well defined.

**FIG 1:**
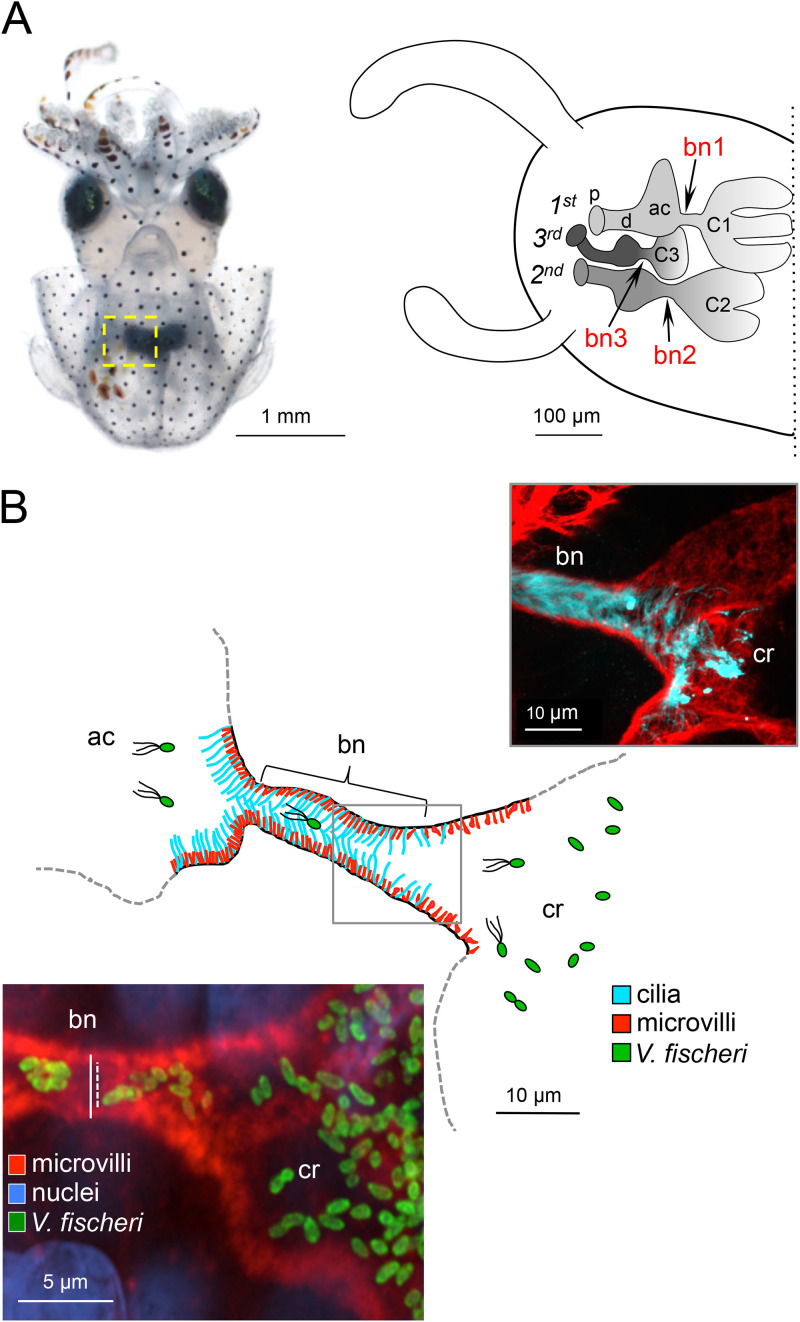
Bacterial symbionts colonize the host by migrating through several tissue microenvironments. (A, left) Light micrograph of an Euprymna scolopes hatchling. The yellow dashed box encloses one half of the ventrally located symbiotic light-emitting organ. (Right) Schematic of the area indicated by the yellow box, illustrating the internal light organ tissue structures with which Vibrio fischeri cells interact during their migration from seawater through three surface pores (p), down a duct (d), across an antechamber (ac), through a bottleneck (bn) and into the associated crypt (cr). The three bottlenecks (bn1 to bn3) are indicated, as well as the order of appearance of the three pathways and corresponding crypts (C1 to C3) during embryogenesis. (B, middle) Schematic of a cross section of the light organ’s tunnel-like bottleneck at the time of colonization; i.e., the first association between bacterial symbionts and previously aposymbiotic tissue. The gray box corresponds to the area in the two accompanying confocal micrographs of a representative bottleneck-crypt transition region. (Right) V. fischeri cells move through lumen with cilia (anti-acetyl alpha tubulin [light blue]) and microvilli (phalloidin [red]). (Left) When the crypts contain dense populations of wild-type V. fischeri (making green fluorescent protein [green]), the bottleneck constricts. Bottleneck measurements were made at the narrowest point of the cross section, from the dense phalloidin-stained terminal web (solid white line = 3.3 μm). Due to the microvilli protruding into the bottleneck lumen, the space available for the migrating V. fischeri was best captured by the dashed white line (2.2 μm).

One of the most conspicuous effects of bidirectional host-symbiont communication (20) is the shaping of the physiological and developmental responses in the microenvironment of host tissues (21–26). In the squid-vibrio system, the host-symbiont dialogue begins with interactions on the light organ surface as the symbiont cells aggregate (27–30). Within hours, V. fischeri cells enter the underlying tissues through pores and proceed down a complex, ∼150-μm migration path (13, 31), a behavior that requires that bacteria are motile and chemotax toward a gradient of host-generated chitobiose (32, 33). Just before entering the blind-ended crypts where they will reside and communicate with the host, the symbionts pass through a bottleneck that constricts dramatically only after they have entered (34, 35). After a period of rapid proliferation, the population of symbionts fills the crypts and persists in a host-associated state, which is characterized by a loss of flagella, slow growth, and high luminescence output (36, 37). Each dawn, 95% of the symbionts are expelled through the pore, and the remaining cells repopulate the crypts (38). In this species-specific symbiosis, only V. fischeri cells are capable of completing the migration and colonizing the crypts and, thus, triggering bottleneck closure. In the bilobed light organ, each side contains a set of three crypts (39) (Fig. 1A). While the major crypt (crypt 1 [C1]) has nearly six times the luminal volume of either of the two minor crypts (C2 and C3) (39), all three crypts are accessed by V. fischeri at the same time following inoculation by bacteria in the seawater (40), and all three of the associated bottleneck tissues constrict in response to colonization (35).

This symbiont-induced constriction occurs within the broader landscape of a series of other bacterium-triggered developmental responses in the host (4, 25, 41, 42) that require metabolically active symbionts. Luminescence seemed to play a role in the bottleneck’s constriction (35), and quorum sensing appeared responsible for controlling the bottleneck’s diameter, thereby defining its proposed “gatekeeper” function (35).

To discover the symbiont cue(s) responsible for driving constriction of these bottlenecks, we colonized the light organ crypts with mutant strains of V. fischeri and assessed the subsequent tissue responses. Specifically, three bacterial factors were manipulated: (i) the synthases (AinS or LuxI) of the two V. fischeri autoinducers (C8 or 3O-C6, respectively), (ii) their shared receptor, the transcriptional regulator LuxR, and (iii) bioluminescence production (16). We found that quorum sensing, mediated by LuxIR, is the most pronounced driver of both bottleneck constriction and successful maintenance of the symbiont population. In addition, we showed that inhibitors of actin polymerization could open the bottleneck, suggesting that this activity underlies the constriction. These findings provide new insight into how signals that coordinate the behavior of symbionts also control a critical symbiotic phenotype of host tissues.

## RESULTS

### The effect of quorum sensing (QS) on bottleneck constriction begins early in symbiotic development.

**FIG 2:**
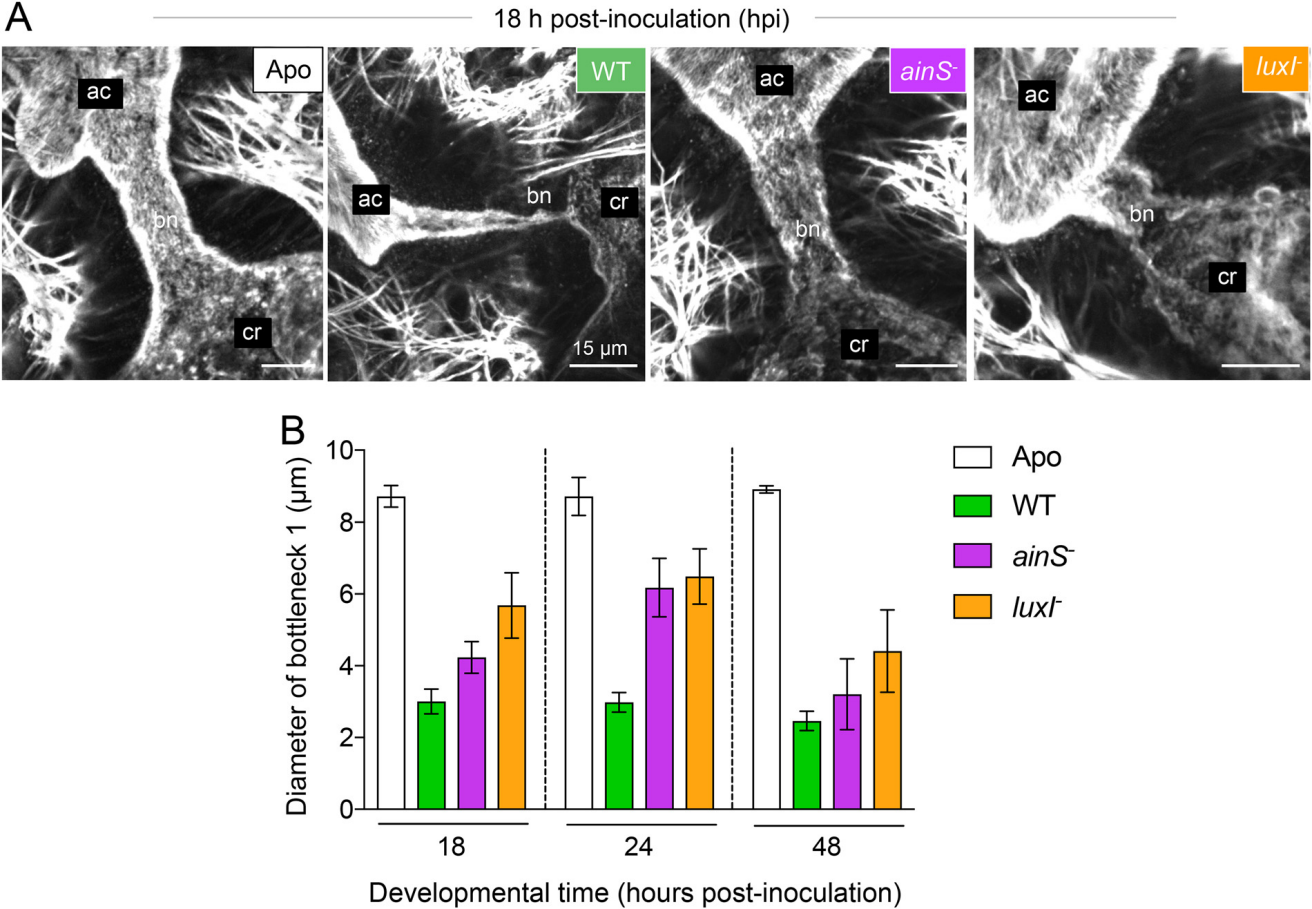
Colonization by quorum-signaling mutants of V. fischeri resulted in abnormal bottleneck constriction. (A) Representative confocal micrographs of the bottleneck (type 1) morphology in response to colonization by wild type (WT) or *ainS*^−^ or *luxI*^−^ mutants of V. fischeri, compared to aposymbiotic (Apo) animals, at 24 h postinoculation (hpi). F-actin (stained by phalloidin) is shown in gray, highlighting the terminal web of the bottleneck epithelium. (B) Levels of bottleneck 1 constriction of colonized organs relative to aposymbiotic (uncolonized) ones when the crypt was colonized by the wild type or *ainS*^−^ or *luxI*^−^ mutants. Bars represent the average bottleneck 1 diameter across all experiments at each of three time points, and error bars indicate the standard errors of the means; see Fig. S3 in the supplemental material for complete data sets. Data were analyzed by a mixed model with a random effect structure for host individual nested in experiment. Strain type and time were added to the model as fixed effects. Comparison of the nested model showed a significant interaction effect of time and strain type on the bottleneck responses, as determined by comparing the models by chi-square test (χ^2^ = 47, df = 6, *P* < 0.0001).

The earliest stage of symbiotic development examined here, 18 h postinoculation (hpi), was after the first venting event and during the daytime regrowth of the symbiont population. At this time, the bottlenecks (Fig. 1B) associated with the most developed crypt type (“major crypt” C1) that were colonized by wild-type (WT) V. fischeri strain ES114 were already ∼65% narrower than their uncolonized (i.e., aposymbiotic [Apo]) counterparts (Fig. 2). This normal response to symbiosis was maintained at 24 h and intensified to ∼75% narrower after 48 h of symbiotic development, while the diameter of Apo bottlenecks remained unchanged and wider than the bottlenecks of WT-colonized crypts over this same interval (Fig. 2B). Although V. fischeri strains carrying null mutations in either of the genes encoding autoinducer synthases (*ainS* or *luxI*) were able to colonize the light organ crypts to normal levels (Tables 1 and 2; see Fig. S1 in the supplemental material), the bottlenecks were dramatically less constricted than those associated with crypts colonized by their WT parent (Fig. 2A). At 24 hpi, (during the animal’s nighttime) when crypts are typically full and functional, the colonization levels of these two QS mutants were often lower than those of WT; however, this reduction, when present, did not correlate with the extent of the defect in the bottleneck’s constriction (Table 2 and Fig. S1). While the host responses to *ainS* and *luxI* mutants were intermediate between WT and Apo bottlenecks at each stage of early development, this defect was more pronounced at the early time points (18 and 24 hpi) (Fig. 2B) and more severe for the *luxI* mutant at all three time points (Table 2). Taken together, these data indicate that the ability of the symbiont to activate QS plays a substantial role in bottleneck constriction.

**TABLE 1:** Bacterial strains used in this study

Strain	Descriptor	Phenotype	Description	Reference
ES114	WT	Wild type	Wild-type *Vibrio fischeri* isolate from *Euprymna scolopes* light organ	94
BDB209	*ainS*^−^ + *ainS*	*ainS* complement	CL21 with *att*Tn*7*::*ainSp-ainS*, Cm^r^	This work
BDB210	*ainSluxI*^−^ + ainS	*ainSluxI*^−^ + *ainS*	CL24 with *att*Tn7::*ainSp-ainS*, Cm^r^ Em^r^	This work
BDB215	*luxI*^−^ + *luxI*	*luxI* complement	VCW2G7 with *att*Tn7::*luxIp-luxI*, Em^r^	This work
BDB216	*ainSluxI*^−^ *+ luxI*	*ainSluxI*^−^ *+ luxI*	CL24 with *att*Tn7::*luxIp-luxI*, Cm^r^ Em^r^	This work
BDB231	*luxR*^−^ + *luxI*	3O-C6 without receptor	CL53 + pVSV105::*luxI*, Cm^r^ Em^r^	This work
BDB242	Δ*luxIR lacZp-lux*	Luminous-positive signaling-negative	Δ*luxIR*::P_A1/O4/O3_; constitutive promoter upstream of *luxCDABEG*	This work
CL21	*ainS* ^−^	*ainS*^−^, no C8	*ainS::cat*, Cm^r^	10
CL24	*ainS* ^−^ *luxI* ^−^	*ainS*^−^*luxI*^−^, no C8 or 3O-C6	*ainS*::*cat*, *luxI* frameshift, Cm^r^	10
CL53	*luxR* ^−^	*luxR*^−^, no receptor for 3O-C6	*luxR*::*ermR*^−^ (VF_A0925), Em^r^	95
EVS102	Δ*luxCDABEG* (Δ*lux*)	Nonluminous	Δ*luxCDABEG* (VF_A0923-0918); *lux* gene-encoding locus	40
SMG28	WT-YFP	WT + pEKCB1	Wild-type ES114, *yfp*, Km^r^	This work
SMG27	WT-CFP	WT + pEKCB2	Wild-type ES114, *cfp*, Km^r^	This work
SMG30	Δ*lux*-YFP	Δ*luxCDABEG* + pEKCB1	EVS102, *yfp*, Km^r^	This work
SMG29	Δ*lux*-CFP	Δ*luxCDABEG* + pEKCB2	EVS102, *cfp*, Km^r^	This work
SMG34	*luxI*^−^-YFP	*luxI*^−^ + pEKCB1	VCW2G7, *yfp*, Km^r^	This work
SMG33	*luxI*^−^-CFP	*luxI*^−^ + pEKCB2	VCW2G7, *cfp*, Km^r^	This work
SMG38	*luxR*^−^-YFP	*luxR*^−^ + pEKCB1	CL53, *yfp*, Km^r^	This work
SMG37	*luxR*^−^-CFP	*luxR*-^−^ + pEKCB2	CL53, *cfp*, Km^r^	This work
SMG36	Δ*luxIR lacZp-lux*-YFP	Δ*luxIR*::P_A1/O4/O3_ + pEKCB1	BDB242, *yfp*, Km^r^	This work
SMG35	Δ*luxIR lacZp-lux*-CFP	Δ*luxIR*::P_A1/O4/O3_ + pEKCB2	BDB242, *cfp*, Km^r^	This work
VCW2G7	*luxI* ^−^	*luxI*^−^, no 3O-C6	*luxI* (VF_A0924) frameshift	10
VCW3F6	*lysA* ^−^	*lysA*; lysine auxotrophy	*lysA*::Tn*kan* (VF_2485)	44
WM3064			*E. coli* conjugation strain; *thrB1004 pro thi rpsL hsdS lacZ*Δ*M15 RP4-1360* Δ(*araBAD*)*567* Δ*dapA1341*::[*erm pir*(wt)]	96

**TABLE 2:** Summary of bottleneck responses

Strain	Bottleneck diam			Total no. of expts different from WT (% of expts)^*c*^	Gene products altered	CFU level (% WT) at 18 hpi; 24 hpi^*d*^
	18 hpi^*a*^ (>5 μm)^*b*^	24 hpi (>5 μm)	48 hpi (>4 μm)			
*ΔluxCDABEG* (*Δlux*)	2/4	3/3	4/5	9/12 (75)	No light	117; 57
*ainS* ^−^	1/4	3/4	1/3	5/11	No C8	82; 94
*ainS*^−^ *+ ainS*	0/4	0/1	0/3	0/8		
*luxI* ^−^	3/5	5/6	3/4	11/15 (73)	No 3O-C6	142; 65
*luxI*^−^ *+ luxI*	0/5	0/3	1/4	1/12		
*ainS* ^−^ *luxI* ^−^	4/4	1/1	1/4	6/9 (67)	No C8 or 3O-C6	78; 36
*ainS*^−^*luxI*^−^ *+ ainS*	4/4	1/1	2/4	7/9 (78)	No 3O-C6	88; 36
*ainS*^−^*luxI*^−^ *+ luxI*	2/4	0/1	0/4	2/9	No C8	115; 48
*luxR* ^−^	2/4	1/2	3/5	6/11 (55)	No regulator	102; 36
*luxR*^−^ *+ luxI*	2/3	1/1	3/4	6/8 (75)	No regulator + 3O-C6	90; 42
*ΔluxIR* * lacZp-luxCDABEG*	2/2	4/4	1/3	7/9 (78)	No regulator or 3O-C6 + light	52; 46

a Time during symbiotic development, hours postinoculation (hpi).

b Criteria to define bottleneck 1 (BN1) response, operationally defined for each time point based on WT. Data are displayed as the number of experiments with criterion/total number scored.

c Each experiment includes 8 to 14 light organ measurements per treatment.

d Level of V. fischeri population as the percentage of CFU/ relative to WT-colonized light organs (see Fig. S1 in the supplemental material for full data set).

10.1128/mBio.02402-21.1 FIG S1 Colonization and luminescence levels of V. fischeri QS mutants during the early stages of inoculation. Strain type, developmental time, as well as the interaction were each assessed for its effect on colonization level in the animal by a two-way ANOVA. Strain type accounts for 13.7% of the total variance (*F*_9,182_ = 3.65; ***, *P *< 0.001), while the contribution of developmental time was not significant. The interaction of the two factors accounts for 9.72% of the variance (*F*_9,182_ = 2.66; **, *P *< 0.01). Using a Tukey’s multiple-comparison test, differences between strains were assessed, and significance was indicated by asterisks as follows: *, *P* < 0.05; **, *P *< 0.01. (A) Colonization levels of each strain at 18 h postinoculation (*n* = 9 animals); bars show standard deviations. (B) Colonization levels of each strain at 24 hpi (*n* = 11 animals). (B’) Relative luminescence of animals colonized by each strain at 24 h postinoculation (CFU counts in panel B), a time of peak wild-type luminescence. Values below 1 light unit (LU) were considered undetectable (dashed line). CFU, colony-forming units; RLU, relative light units. Download FIG S1, TIF file, 0.5 MB. Copyright © 2021 Essock-Burns et al. 2021 Essock-Burns et al. https://creativecommons.org/licenses/by/4.0/ This content is distributed under the terms of the Creative Commons Attribution 4.0 International license.

10.1128/mBio.02402-21.3 FIG S3 Total data set showing the box plots for all bottleneck responses to V. fischeri strains over the course of early symbiotic development. (A to C) Animals that were 18 h postinoculation (A), 24 h postinoculation (B), and 48 h postinoculation (C). The dashed gray line indicates the upper cutoff for WT-like diameter constriction responses, 5 μm at 18 and 24 hpi and 4 μm at 48 hpi. Colors and number indicate different experiments; each bar represents 8 to 14 light organ measurements. Download FIG S3, TIF file, 0.6 MB. Copyright © 2021 Essock-Burns et al. 2021 Essock-Burns et al. https://creativecommons.org/licenses/by/4.0/ This content is distributed under the terms of the Creative Commons Attribution 4.0 International license.

### QS, but not one of its products, luminescence, is crucial for bottleneck constriction.

**FIG 3:**
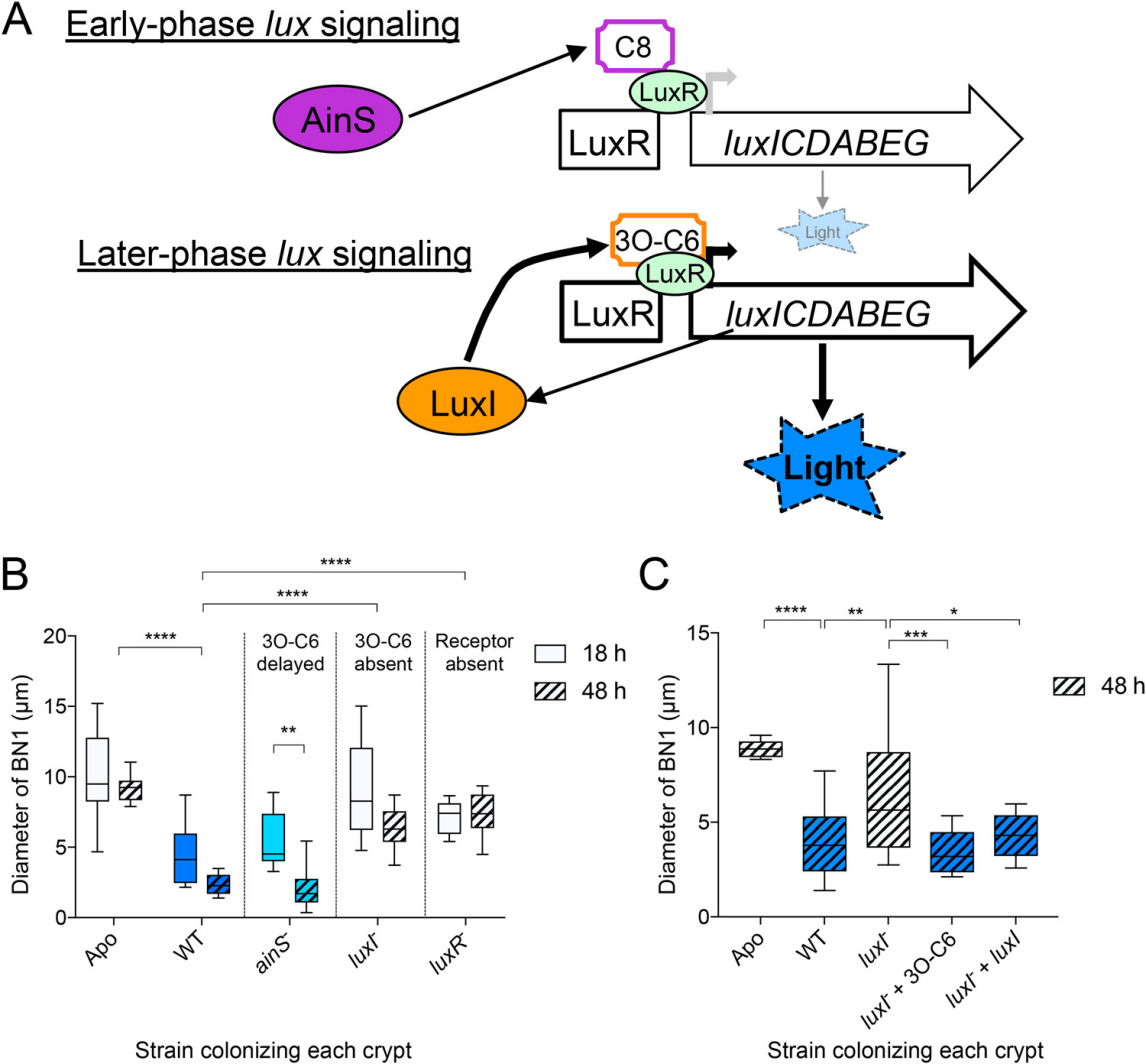
Later-phase LuxIR quorum sensing has a strong impact on host bottleneck constriction. (A) Schematic of quorum sensing (QS) once V. fischeri colonizes the host light organ, focusing on the *luxIR*-encoded signaling that drives symbiotic light production, i.e., *lux* gene expression. In early phase signaling, V. fischeri produces only the *ainS*-encoded QS molecule (C8), which binds poorly to the low level of uninduced LuxR, resulting in some luminescence, but no LuxIR signaling. After the symbiont population has filled the organ and QS has progressed to the later phase, *luxR* is induced, increasing the amount of LuxR and subsequently, of the *luxI*-encoded signal, 3O-C6, which binds well to the cognate LuxR, resulting in a strong induction not only of the *lux* operon (and subsequently, bioluminescence) but also the expression of other LuxIR-responsive genes (11). (B) Bottleneck responses to colonization by strains defective in QS genes, leading to either delayed or absent 3O-C6 production (i.e., *ainS* and *luxI* mutants, respectively) or to their detection (i.e., by the transcriptional regulator-encoding *luxR*) are shown at 18 and 24 hpi. Bars represent 8 to 10 measurements of bottleneck type 1 (BN1); the intensity of blue indicates the relative level of light output of animals colonized by these strains (Fig. S4A’). Using a two-way analysis of variance (ANOVA), all three factors (strain, colonization time, and their interaction) contributed significantly to the percentage of total variation observed. Strain type contributed 54% (*F*_4, 84_ = 34, *P* < 0.0001), colonization time contributed 7.4% (*F*_1, 84_ = 19, *P* < 0.0001), and their interaction contributed 4.2% (*F*_4, 84_  = 2.6, *P* < 0.05). A Dunnett’s multiple-comparison test was used to assess differences between groups. The top three sets of asterisks indicate differences between treatments at both time points; the bottom set shows a comparison within a mutant at the two time points. (C) Pharmacological and genetic complementation of *luxI* function at 48 h after inoculation. Bars represent 9 to 14 BN1 measurements; the animals with detectable luminescence are showin in blue (Fig. S2B). A one-way ANOVA and Tukey’s *post hoc* test were used to compare responses to strains (*F*_8, 87_ = 9.0, *P* < 0.0001). Values that are significantly different are indicated as follows: *, *P* < 0.05, **, *P* < 0.01, ***, *P* < 0.001, ****, *P* < 0.0001. Not shown on the graph was the finding that both genetic and pharmacological complementation of the *luxI*^−^ mutant were significantly different from the aposymbiotic (****).

In the early phase of QS, as V. fischeri colonizes the crypts, the symbionts pass through an early C8-dominated phase, followed by a 3O-C6-dominated one (Fig. 3A). We compared early (18 hpi) and late (48 hpi) symbiotic responses to V. fischeri strains with mutations in genes encoding the synthases of these autoinducers (*ainS* or *luxI*) or the LuxR regulator (*luxR*). While the *ainS* mutant (CL21 [Table 1]) exhibited a mild defect early, by 48 h into symbiotic development, the constriction had reached the normal, WT level (Fig. 3B, Fig. S2A, S3A, and S3C, and Table 2). In contrast, the bottleneck defect in response to the *luxI* mutant (VCW2G7 [Table 1]) was significantly greater at 48 hpi (Fig. 3B). This *luxI* mutant defect was abolished at 48 h if the LuxI product (3O-C6) was added either directly or through genetic complementation (i.e., by carriage of a multicopy plasmid constitutively expressing a wild-type copy of *luxI*; BDB215 [Table 1]) (Fig. 3C and Fig. S2B). While at 24 hpi the bottleneck defect associated with colonization by the *ainS* mutant was comparable to that of the *luxI* mutant, *luxI* complementation of the double mutant (*ainS*^−^ *luxI*^−^; BDB216 [Table 1]) was more effective at abrogating this phenotype (Table 2 and Fig. S3), probably because the *ainS* defect is upstream of *luxI* and becomes irrelevant after late-phase signaling dominates (Fig. 3A).

10.1128/mBio.02402-21.2 FIG S2 Diameter of bottleneck type 1 (BN1) in response to colonization by V. fischeri mutants at 18 and 48 h postinoculation (hpi) (left) and the associated levels of animal luminescence (right). (A) Left, bars represent 8 to 10 light organ measurements per treatment, and the values were compared by two-way ANOVA for (i) colonization time (*F*_1, 164_  = 39.6; *P *< 0.0001), (ii) colonizing strain (*F*_9, 164_  = 25.5; *P *< 0.0001), and (iii) interaction between these two variables (*F*_9, 164_ = 2.34; *P = *0.017). Multiple *t* tests were used to compare each time point by treatment: *ainS*^−^ (*t* = 4.42, df = 17; *P* < 0.01); *ainS*^−^ + *ainS* (*t* = 8.83, df = 16; *P *< 0.0001); *ainS*^−^ *luxI*^−^ + *luxI* (*t* = 4.02, df = 16; *P* < 0.01). (Right) Luminescence of animals; *N* = 5 to 8 animals; values less than 1 light unit (LU) were below the limit of detection (dashed line). (B, left) Bars represent 9 to 14 light organ measurements per treatment, and the values were compared by two-way ANOVA for (i) colonization time (*F*_1, 195_ = 0.04; *P* = 0.845), (ii) colonizing strain (*F*_8, 195_ = 15.8; *P* < 0.0001), and (iii) interaction (*F*_8, 164_  = 3.27; *P* = 0.0016). A Sidak’s multiple-comparison test was used to examine the overall strain effects on the bottleneck diameter. (Right) Luminescence of animals; *N* = 5 to 8 animals; values below the dashed line were under the limit of detection. Error bars indicate the standard deviations. Where indicated, the values comparing treatments were significantly different and indicated by asterisks as follows: *, *P < *0.05; **, *P < *0.01; ***, *P < *0.001; ****, *P < *0.0001. Download FIG S2, TIF file, 0.6 MB. Copyright © 2021 Essock-Burns et al. 2021 Essock-Burns et al. https://creativecommons.org/licenses/by/4.0/ This content is distributed under the terms of the Creative Commons Attribution 4.0 International license.

10.1128/mBio.02402-21.4 FIG S4 Effect of pharmacological addition of 3O-C6 on colonization characteristics of V. fischeri mutants after 24 hpi. (A) Bottleneck diameter. Blue data points indicate animals that were detectably luminescent (*N* = 12 to 18 light organ measurements per treatment). A one-way ANOVA and Tukey’s *post hoc* test were used (*F*_11, 151_  = 4.682; *P* < 0.0001). (A’) Luminescence of animals. Values less than 1 light unit were below the limit of detection (dashed line). (*N* = 6 to 9 animals per treatment). Error bars indicate the standard deviations. Where indicated, the comparisons between treatments were significantly different with the following *P* values: *, *P < *0.05; **, *P < *0.01; ***, *P < *0.001. Download FIG S4, TIF file, 0.4 MB. Copyright © 2021 Essock-Burns et al. 2021 Essock-Burns et al. https://creativecommons.org/licenses/by/4.0/ This content is distributed under the terms of the Creative Commons Attribution 4.0 International license.

Because QS complementation restored both bottleneck constriction and the luminescence of otherwise “dark” *luxI* mutant colonized animals to wild-type levels (Fig. S4A’), we sought to determine whether symbiotic light production was, itself, the key driver of constriction. This hypothesis was based on two prior reports of bioluminescence inducing host tissue responses: (i) driving maturation of the crypt epithelium (4, 40) and (ii) regulating the expression of light-sensing genes in the light organ (19, 43). Because some *ainS* mutant-colonized animals were bioluminescent while others were not, one approach was to compare the bottleneck responses of these two sets of animals (Fig. S4). The range of the bottleneck defect in crypts colonized by the *ainS* mutant was as wide as those of a dark mutant (Δ*luxCDABEG*) (Fig. S4A), and detectably luminescent animals (blue squares in Fig. S4A) were associated with bottleneck diameters throughout the range, suggesting that luminescence levels alone do not invoke constriction. A second approach was to colonize animals with an auxotrophic mutant (*lysA*^−^) that is unable to synthesize lysine and, thus, reaches a lower level of colonization that, like Δ*luxCDABEG*, is only ∼10% that of the wild type at 48 h (44). While they produced an order of magnitude lower luminescence (Fig. S5A’), colonization by this *lysA* mutant (VCW3F6 [Table 1]) produced a WT level of constriction. Thus, these data provide evidence that a typical level of luminescence emission by the symbionts is neither necessary nor sufficient to induce bottleneck constriction (Fig. S5).

10.1128/mBio.02402-21.5 FIG S5 Effect of symbiont luminescence on bottleneck diameter. (A) Bottleneck (BN) closure in response to mutant strains as a function of their light production. Animals colonized by some mutants were grouped as producing either less than or more than 2 or 20 light units (LU). One light unit was the limit of detection. (A’) Luminescence of animals corresponding to treatments in panel A. (B) Effect of the addition of 3O-C6 on BN diameter. Some animals were measured after treatment with chloramphenicol to remove the symbiont population (cured). (C) Growth rates and yields of strain Δ*luxIR lacZp-lux* were similar to those of the wild type (WT) in either LBS (top) or SWT (bottom) media. Data represent the means from three biological replicates ± 1 standard deviation. (D) In culture, the maximum specific luminescence levels of Δ*luxIR lacZp-lux* were higher than WT. Data are from three biological replicates. OD, optical density at 600 nm. Download FIG S5, TIF file, 0.5 MB. Copyright © 2021 Essock-Burns et al. 2021 Essock-Burns et al. https://creativecommons.org/licenses/by/4.0/ This content is distributed under the terms of the Creative Commons Attribution 4.0 International license.

### Normal bottleneck constriction requires both 3O-C6 signal production and detection.

**FIG 4:**
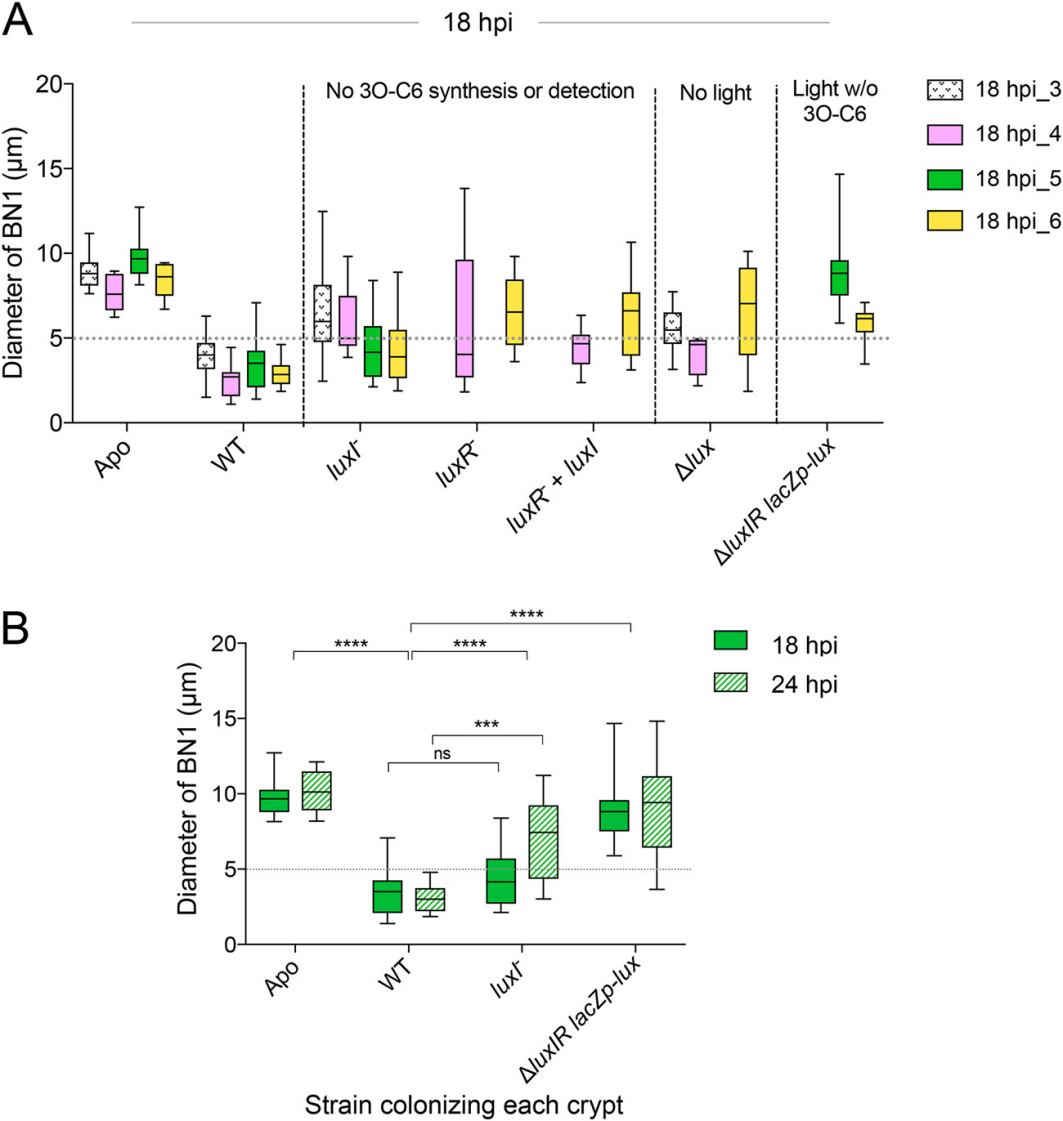
LuxIR activity, independent of bioluminescence, was required for normal bottleneck constriction. (A) The impact on the bottleneck type 1 (BN1) diameter at 18 h postinoculation (hpi), when the associated crypts are colonized by mutants defective in either 3O-C6 synthesis (i.e., *luxI*^−^) or 3O-C6 detection (i.e., *luxR*^−^) or in light production (Δ*lux*) or a combination (i.e., Δ*luxIR lacZp-lux*, referred to as the signaling mutant [see Table 1]). Box plots represent measurements from 8 to 12 light organs; each color indicates one of four independent experiments (see Fig. S3A for additional data). Median values above the dashed line were considered to be different from the response to the wild type (WT). w/o, without. (B) Bottleneck responses measured at two early points in symbiotic development (i.e., 18 and 24 hpi) differed from WT when the symbionts lacked either *luxI* alone or in combination with *luxR*. Data were compared using a two-way ANOVA, and the strain was the most predictive factor (*F*_3, 86_ = 50; ****, *P* < 0.0001). Using a Tukey’s multiple-comparison test, differences between strains were assessed, and significance was indicated as follows: ***, *P* < 0.001, ****, *P* < 0.0001; ns, not significant. The top set of asterisks indicates differences between treatments at both time points; the bottom set shows comparisons between the WT and the *luxI* mutant at each time point. Data represent 10 to 12 measurements of each condition. For clarity, 24-hpi data are shown with a hatched pattern (see “18 hpi_5” in Fig. S3A and “24 hpi_5” in Fig. S3B for additional data).

Next, we aimed to separate the role of light as an effector of the bottleneck response from that of the LuxIR system (i.e., 3O-C6 signaling), which directly induces luminescence (Fig. 3A). Crypts colonized by symbionts lacking the ability to produce either 3O-C6 (i.e., the *luxI* mutant) or its cognate transcriptional regulator, LuxR (CL53 [Table 1]), typically had abnormal bottlenecks early (i.e., 18 hpi) into symbiotic development (Fig. 4A) that were as poorly constricted as those of a dark symbiont (Δ*luxCDABEG*; EVS102 [Table 1]). Because a *luxIR* mutant is also dark, we then asked whether experimentally restoring its light production could reverse the defect of this mutant; specifically, we constructed a Δ*luxIR* strain in which *luxCDABEG* expression was independent of QS and, instead, was controlled by a constitutive *lac* promoter (i.e., Δ*luxIR lacZp-luxCDABEG*; strain BDB242 [Table 1]). This mutant was brighter than the WT in culture (Fig. S5C and S5D) and colonized to levels within the range of WT (Fig. S1) and produced light in the animal (Fig. S4A’ and S5A’), albeit less than WT. Interestingly, the presence of this luminescence did not improve the bottleneck defect of the *luxIR* signaling mutant (Fig. 4 and Fig. S1, S4A, and S6). As soon as 18 hpi, crypts colonized by the *luxI* mutant strain would sometimes exhibit WT-like bottlenecks (Fig. 4); however, even though it produced light at both early time points (18 and 24 hpi), the Δ*luxIR lacZp-luxCDABEG* strain defect was stronger and failed to elicit a bottleneck constriction response that was different from Apo (Fig. 4 and Fig. S3A and S3B). At the earliest time point examined (18 hpi), the Δ*luxIR lacZp-luxCDABEG* strain has a significantly lower colonization level than the *luxI* mutant (Fig. S1). However, both strains induce an abnormal host response, indicating that the defect is not related to symbiont population size.

10.1128/mBio.02402-21.6 FIG S6 Coinoculation of animals with the LuxIR signaling (Δ*luxIR lacZp-lux*) mutant (see strain BDB242 in Table 1) in combination with the WT or the dark mutant (Δ*luxCDABEG*). (A) The relative occurrence of coinoculated strains colonizing each crypt type (C1 to C3) after 24 or 48 h of symbiotic development. Some crypts contained both strains (WT and Δ*luxIR lacZp-lux*) or neither. (A’) Diameters for the three bottleneck types (BN1 to BN3) when the associated crypt was colonized by either one or both strains of a coinoculation. “Neither” indicates crypts that had neither strain, either because they were uncolonized crypts in symbiotic light organs or because they were aposymbiotic animals. (B) Early (24 hpi [left]) differences in bottlenecks associated with major crypts (BN1) versus minor crypts (BN3) when colonized by each mutant were no longer apparent later in symbiotic development (48 hpi [right]). The factors of crypt contents, bottleneck type, and the interaction were assessed for their contribution to bottleneck constriction over time by a two-way ANOVA and further compared by a Tukey’s *post hoc* test. For the 24 hpi bottleneck responses, bottleneck type accounts for 4.4% of the total variance (*F*_2, 205_ = 6.22, *P < *0.01), while crypt contents account for 3.17% of the total variance (*F*_3, 205_ = 2.95, *P < *0.05). At 48 hpi, crypt contents alone explain 29.9% of the total variance (*F*_3, 178_ = 26.89, *P* < 0.0001). Values that are significantly different are indicated by asterisks as follows: **, *P < *0.01; ***, *P *< 0.001; ****, *P* < 0.0001. Download FIG S6, TIF file, 0.6 MB. Copyright © 2021 Essock-Burns et al. 2021 Essock-Burns et al. https://creativecommons.org/licenses/by/4.0/ This content is distributed under the terms of the Creative Commons Attribution 4.0 International license.

Finally, pharmacological addition of the *luxI*-encoded product, 3O-C6, was not sufficient to induce bottleneck closure in either uncolonized or formerly colonized animals (Fig. S5B), indicating metabolic activity in response is required. Similarly, genetic complementation of V. fischeri mutants with a constituently expressed WT copy of *luxI* on a multicopy plasmid abrogated the bottleneck defect only when LuxR, the functional receptor for 3O-C6 was present (i.e., in “*luxI*^−^ + *luxI*,” but not in “*luxR*^−^ + *luxI*”) (Fig. 3C and 4A and Fig. S3). Taken together, these data suggest that LuxIR signaling, requiring both the *luxI*-encoded autoinducer, 3O-C6, and its cognate transcriptional regulator LuxR, is more critical for bottleneck constriction than the symbionts’ production of light.

### Colonization competition is more affected by the loss of QS than the loss of luminescence.

**FIG 5:**
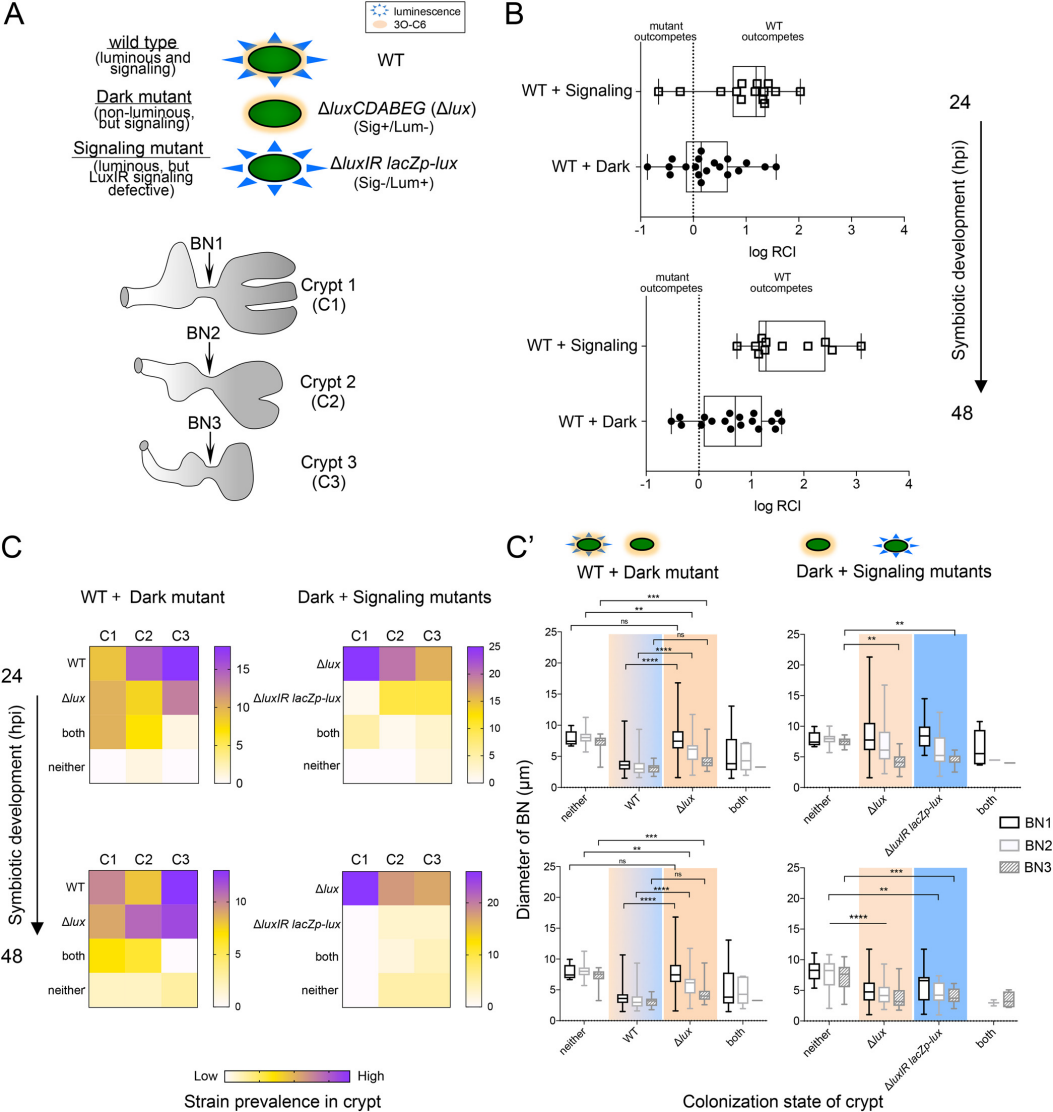
Even when luminescent, an inability to signal via LuxIR yields uncompetitive symbionts unable to initiate the bottleneck response. (A) Schematic of the characteristics of three strains used in pairwise combinations to inoculate light organs, as well as the three types of crypts (C1 to C3) that were colonized and associated bottlenecks (BN1 to BN3; arrows). (B) Relative competitive colonization success of each strain at an early and later stage of symbiotic development. Open squares depict data from animals cocolonized with WT and the luminous signaling-negative (Sig^−^)/luminescence-positive (Lum^+^) mutant (Δ*luxIR lacZp-lux*), and filled circles are those cocolonized with WT and the dark mutant (Δ*lux*). Between 11 and 19 of the 20 animals were inoculated per treatment (top, 24 hpi; bottom, 48 hpi). RCI, relative competitive index. (C) The relative occurrence of the coinoculated strains occupying each crypt type (C1 to C3) over the first 2 days of symbiotic development (i.e., actual number of crypts with that strain or combination). For 24-h animals (*n* = 16 animals with bilobed light organs, i.e., 32 measurements of C1 to C3); 48-h animals (*n* = 14 animals, 28 measurements of C1 to C3). Some crypts contained either both strains or neither (see Fig. S6 for additional data). (C’) Diameters for BN1 to BN3 when the associated crypt was colonized by neither, one, or both strains of a coinoculation. Occurrences with two strains within a single crypt were rare and excluded from statistical analyses. Data were analyzed using a two-way ANOVA to compare the contribution of crypt contents to that of bottleneck type and the interaction. Crypt contents best explained the variance in combinations with WT (between 32 and 52% of the variance), regardless of symbiotic development time. The variance in the combination of dark (Δ*lux*) and Sig^−^/Lum^+^ (Δ*luxIR lacZp-lux*) mutants was better explained (4.4%) by bottleneck type (*F*_2, 205_ = 6.2, *P* < 0.01) than by crypt contents (3.2%) (*F*_3, 205_ = 3.0, *P* < 0.05). A Tukey’s multiple-comparison test was used to distinguish between groups further. Values that are significantly different are indicated by asterisks as follows: **, *P* < 0.01; ***, *P* < 0.001; ****, *P* < 0.0001. Values that are not significantly different (ns) are indicated. “Neither” indicates crypts that had neither strain, either because they were uncolonized crypts in symbiotic light organs or because they were aposymbiotic controls (see Fig. S8 for additional data on this distinction).

Next, we assessed the colonization success of two mutants with opposite functionality in terms of QS and light production, comparing the dark mutant with signaling functionality (Δ*luxCDABEG*), to the *luxIR* signaling mutant that produces light (i.e., Δ*luxIR lacZp-luxCDABEG*). Specifically, we coinoculated newly hatched squid with pairs of three strains: WT, Δ*luxCDABEG*, or Δ*luxIR lacZp-luxCDABEG* (Fig. 5A), each carrying plasmids with different fluorescent markers (Table 3). The hosts were then examined for (i) the light organ population levels of each strain (Fig. 5B), (ii) the localization of each strain in the three crypt types (Fig. 5C and Fig. S6A), and (iii) the response of each bottleneck to the presence of a particular strain (Fig. 5C’ and Fig. S6A’). While the results presented to this point have focused only on constriction of the bottleneck of crypt 1, which is the most mature and houses the majority of symbionts (“major crypt” C1), in this experiment, bottlenecks of all three of the crypt types were examined; i.e., including the “minor crypts,” C2 and C3 (Fig. 1A). We first determined that the Δ*luxIR lacZp*-*luxCDABEG* strain colonizes to a lesser extent than WT at 24 hpi; however, it is not significantly different from the Δ*luxCDABEG* strain (Fig. S1B). In addition, this lower colonization level does not correlate to an inability to occupy specific crypt types. Empty crypts in monocolonized animals were very rarely observed (WT, 1/48 total crypts; Δ*luxCDABEG* mutant, 0/48; Δ*luxIR lacZp*-*luxCDABEG* mutant, 2/42) and were exclusively in C3. Taken together, these points suggest that all three strains can colonize each type of crypt and, thus, can be directly compared in the cocolonization experiments described below.

**TABLE 3:** Plasmids used in this study

Plasmid	Description	Reference or source
pBDB1	pEVS107::*luxI*	This work
pBDB2	pEVS107::*ainS*	This work
pBDB3	pVSV105::*luxI*, 15 bp upstream, 9 bp downstream	This work
pBDB4	pSMV3 with *luxIR* flanking sequences surrounding P_A1/O4/O3_	This work
pEKCB1	Strain tagging, pVSV102 backbone, Km^r^, *yfp*	E. Koch and C. Bongrand
pEKCB2	Strain tagging, pVSV102 backbone, Km^r^, *cfp*	E. Koch and C. Bongrand
pEVS104	Conjugation helper	36
pEVS107	Mini-Tn7 vector; Km^r^ Em^r^	88
pSMV3	Deletion vector, Km^r^, *sacB*	96, 97
pUX-BF13	*tnsABCDE* transposase vector	98
pVSV102	Strain tagging, Km^r^, *gfp*	99
pVSV105	Cloning vector, Cm^r^	99

10.1128/mBio.02402-21.8 FIG S8 The total potential input to a bottleneck response and the primary driver of focal crypt activity. (A) Diameters of all bottleneck types (BN1 to BN3) remained unchanged over development in aposymbiotic animals. (A’) Red points indicate the diameter of bottlenecks without symbionts in the associated crypt, but in an otherwise symbiotic light organ; gray points correspond to aposymbiotic animals in panel A. (B) Bottleneck responses to strains occupying crypt 1 in coinoculated squid with the *luxI* mutant and Δ*lux* strain compared to empty crypts, whether within aposymbiotic light organ or an empty crypt within a light organ with symbionts in neighboring crypts (red points). (B’) Animal luminescence when colonized by each strain or combination of strains. Points below the dashed line were considered below the limit of detection. Download FIG S8, TIF file, 0.4 MB. Copyright © 2021 Essock-Burns et al. 2021 Essock-Burns et al. https://creativecommons.org/licenses/by/4.0/ This content is distributed under the terms of the Creative Commons Attribution 4.0 International license.

When summed across the entire light organ, the luminescent signaling mutant (Δ*luxIR lacZp*-*luxCDABEG*) performed more poorly than the dark mutant (Δ*luxCDABEG*) in competitions with WT, and this defect worsened between 24 and 48 hpi (Fig. 5B and Fig. S6). A closer examination of the prevalence of each strain in individual crypts revealed that the signaling mutant was outcompeted for crypt 1 by both WT (Fig. S6A) and the dark mutant (Fig. 5C). Similar to results of earlier studies (45, 46), in coinoculations of WT and Δ*luxCDABEG* strains, the dark mutant was most successful in the less mature crypts, particularly crypt 3 where it was nearly as prevalent as WT (Fig. 5C). Similarly, the frequency of crypts that contained both WT and a mutant strain together was much greater for coinoculations including the dark mutant (Δ*luxCDABEG*) (Fig. 5C) than those with the signaling mutant (Δ*luxIR lacZp*-*luxCDABEG*) (Fig. S6A). Thus, the absence of LuxIR signaling resulted in the most severe competitive defects. When we compared the response of bottlenecks associated with crypt 1 (most mature) to crypt 3 (least mature), we saw that the least mature gatekeeper was less discriminant; i.e., it responded to each mutant no differently than it did to WT at both 24 and 48 hpi (Fig. S6B). The response of the most mature bottleneck of crypt 1 (BN1), or the ability to discriminant symbiont activity, is muted by 48 hpi for both mutants (Fig. 5C). These findings suggest that either the activity of the symbionts in the minor crypts, or the mechanism inducing bottleneck constriction, may be different depending on the degree of maturation of the tissue.

We next sought to determine whether the colonization defect observed in the signaling mutant was due to a priority effect caused by a slower migration rate (47). That is, if the WT and the dark mutant colonize more quickly, they might impede any subsequent occupation by the signaling mutant if it were to reach the crypts more slowly. To test this hypothesis, we inoculated juveniles first with the signaling mutant (Δ*luxIR lacZp*-*luxCDABEG*) and then, after 3 h, with a second, competing strain. The second strain (expressing a distinguishing fluorescent label) was WT, the dark mutant, or an additional dose of the signaling mutant (Fig. S7 and Table 1). We predicted that, if the signaling mutant were being outcompeted because it was slower to reach the crypts, the 3-h head start should eliminate its colonization defect relative to the secondarily inoculated strain. However, the addition of the second strain lowered both the total CFU and the relative competitive index of the signaling mutant (Fig. S7), suggesting that the signaling mutant’s defect results from strain-to-strain processes within the crypts rather than a difference in the rate at which each strain accesses the crypt.

10.1128/mBio.02402-21.7 FIG S7 The LuxIR signaling mutant that produces light (Δ*luxIR lacZp-lux*; Sig^−^/Lum^+^) colonized more poorly than a dark mutant (Δ*lux*) at 24 hpi. (A) Relative competitive index of signaling mutant when it was inoculated 3 h before a second, competing strain was added. (B) Total CFU of Δ*luxIR lacZp-lux* in light organs when a second strain was present. Kruskal-Wallis and Dunn’s *post hoc* tests were used (*H *= 8.84, df = 29, *P < *0.05). Where indicated, the comparisons between treatments were significantly different (**, *P <* 0.01). Download FIG S7, TIF file, 0.3 MB. Copyright © 2021 Essock-Burns et al. 2021 Essock-Burns et al. https://creativecommons.org/licenses/by/4.0/ This content is distributed under the terms of the Creative Commons Attribution 4.0 International license.

### While QS activity within the associated crypt dominates, a bottleneck’s response is also influenced by QS occurring in neighboring crypts.

To determine whether the response of a given bottleneck is dictated solely by the strain colonizing the associated crypt or whether there is also input from nearby crypts, we compared the bottleneck diameters of crypts of Apo animals (i.e., no bacteria introduced at all) to those associated with uncolonized crypts within an otherwise symbiotic (Sym) light organ (i.e., when nearby crypts were colonized) (Fig. S8A). Although empty crypts are rare when WT cells are in the inoculum, they were observed 38 times, and in most cases (58%), the bottleneck was more closed in instances of uncolonized crypts in otherwise Sym animals (red points in Fig. S8A’) than in Apo crypts. These data indicate that bottleneck closure is, at least in part, influenced by the colonization state of other crypts in the light organ.

We next asked whether this tendency of empty crypts in an otherwise colonized light organ to have narrower bottlenecks might be due to signaling from the symbionts in a neighboring crypt. To test this hypothesis, we used a mixed model to predict that, if a neighboring crypt contains nonsignaling symbionts (e.g., Δ*luxIR lacZp*-*luxCDABEG*), the bottleneck of an empty crypt will remain open (∼8 μm); however, if the neighboring crypt contains symbionts with a functional LuxIR (e.g., colonized by WT or Δ*luxCDABEG*), then the bottleneck will be narrower (see Text S1 in the supplemental material). Analyses of these conditions provided evidence that this intercrypt effect did occur and that a V. fischeri population with LuxIR activity in a crypt has some, albeit incomplete, influence on a neighboring bottleneck (χ^2^  = 5.4, df = 1, *P* = 0.02). When we tested models that took the crypt identity (C1, C2, or C3) into account or classified them as major (C1) or minor (C2/C3), the model could no longer explain the neighbor effect. Taken together, these results indicated that LuxIR signaling within a given crypt exerts some influence on nearby bottleneck tissue.

10.1128/mBio.02402-21.10 TEXT S1 Supplemental methods. Extended methods containing a detailed explanation of genetic constructions, microscopy imaging, and mathematical modeling approaches. Download Text S1, PDF file, 0.07 MB. Copyright © 2021 Essock-Burns et al. 2021 Essock-Burns et al. https://creativecommons.org/licenses/by/4.0/ This content is distributed under the terms of the Creative Commons Attribution 4.0 International license.

Finally, we asked whether both strains that produce light (WT and Δ*luxIR lacZp*-*luxCDABEG*) have an equal effect on the bottleneck or, instead, in the absence of *luxIR* signaling, even in the presence of luminescence, there was a significantly diminished response. To address this question, we tested a three-tiered score that differentiated between the two luminous strains (WT and Δ*luxIR lacZp*-*luxCDABEG*), as well as a model that combined both light-producing strains together (Text S1). The former model (three-tiered score of light emission) best predicted the effect on the bottleneck, emphasizing that symbiont bioluminescence alone did not restore the bottleneck response in a signaling mutant (ΔAIC [Akaike information criterion] = 0.0, df = 7, weight = 0.93). Taken together, these data suggest that the activity of LuxR-regulated genes can have consequences on host tissue beyond the adjacent bottleneck of a given crypt.

### QS-mediated bottleneck constriction requires actin polymerization.

**FIG 6:**
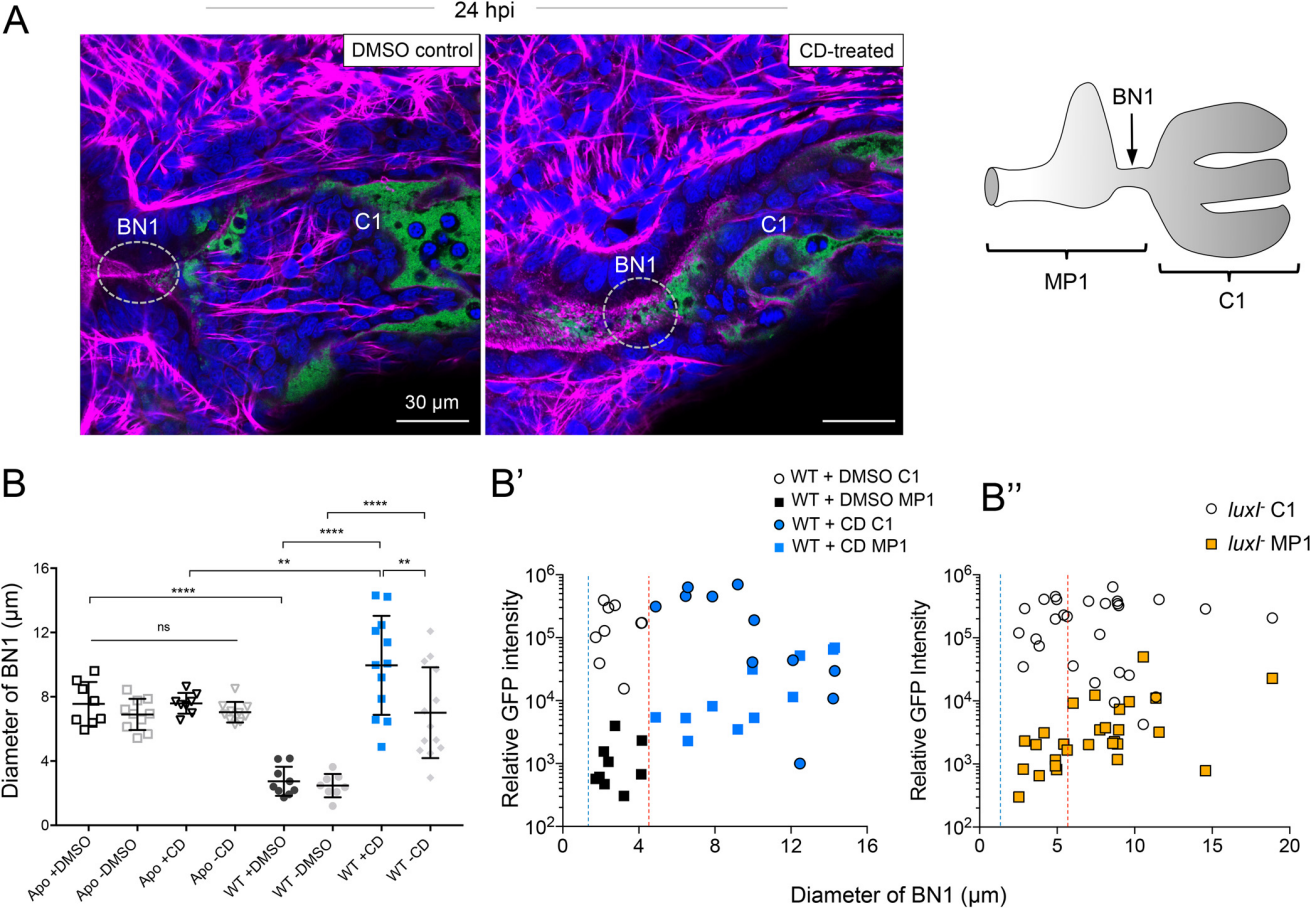
Inhibition of actin polymerization reversibly disrupted symbiosis-triggered bottleneck constriction and gatekeeper function. (A) Confocal micrographs of symbiotic light organs colonized by WT V. fischeri expressing green fluorescent protein (GFP [green]); the associated BN1 (dashed gray oval) was visualized by phalloidin staining of the F-actin terminal web (magenta). Host nuclei and hemocytes were stained with TO-PRO-3 (blue). (Left) Unperturbed/constricted bottleneck of a dimethyl sulfoxide (DMSO)-treated solvent control. (Middle) Perturbed/widened bottleneck resulting from treatment with the actin polymerization inhibitor, cytochalasin D (CD) (see Fig. S9). (Right) Diagram outlines the two luminal microenvironments that the bottleneck bridges as reference for the sites of symbiont position shown in panels B’ and B’’. (B) The diameter of BN1 in response to either a 3-h CD treatment (+CD) prior to the endpoint of the colonization or 3 h after its subsequent relief in animals rinsed to remove the inhibitor (-CD). Data were analyzed using a one-way ANOVA and Tukey’s *post hoc* test (*F*_7, 70_ = 18, *P* < 0.001). Values that are significantly different are indicated by asterisks as follows: **, *P* < 0.01; ****, *P* < 0.0001. Values that are not significantly different (ns) are indicated. (B’) The abundance of V. fischeri cells (based on relative GFP intensity) at 24 h postinoculation (hpi) within two tissue regions: migration path 1 (MP1) including the duct, antechamber and bottleneck, and crypt 1 (C1) as a function of bottleneck diameter (panel A, right). Data were analyzed by a Pearson’s correlation between diameter of the bottleneck and GFP intensity (proxy for V. fischeri abundance). Black points correspond to DMSO treatment (solvent control), and blue points correspond to CD treatment. The blue dashed line shows the lower limit, and the red dashed line shows the upper limit of the bottleneck diameter measured in response to colonization by wild type V. fischeri. (B’’) The relative position of the *luxI*^−^ strain of V. fischeri at 24 hpi. These data represent measurements of GFP fluorescence and BN diameter from three independent experiments; each symbol shows the value for a single light organ set.

Because the bottleneck response to symbiosis resembled typical cytoskeletal remodeling behavior in eukaryotic cells, we asked whether closure was mediated by host actin polymerization. Specifically, we incubated the animals with a reversible inhibitor of actin polymerization, cytochalasin D (CD) (48), and found that the constriction of symbiotic bottlenecks was relieved, and they became at least as open as Apo ones (Fig. 6 and Fig. S9). Similar results were found with another polymerization inhibitor, CK666, which alters actin polymerization via the Arp2/3 complex (49) (Fig. S9B).

10.1128/mBio.02402-21.9 FIG S9 Symbiotic BN1 diameter responses at 18 h postinoculation (hpi) when actin polymerization was inhibited. (A) Constriction was suppressed in a dose-dependent manner when the polymerization inhibitor cytochalasin D (CD) is present. CD was added to seawater containing WT-colonized animals for 3 h before measuring their bottleneck diameters. (B) Bottleneck responses of light organs colonized by different strains when actin polymerization was inhibited by the addition of either CD or a low or high concentration of another inhibitor, CK666, an Arp2/3 complex actin polymerization inhibitor. Dimethyl sulfoxide (DMSO) was used as the solvent control. Download FIG S9, TIF file, 0.4 MB. Copyright © 2021 Essock-Burns et al. 2021 Essock-Burns et al. https://creativecommons.org/licenses/by/4.0/ This content is distributed under the terms of the Creative Commons Attribution 4.0 International license.

**FIG 7:**
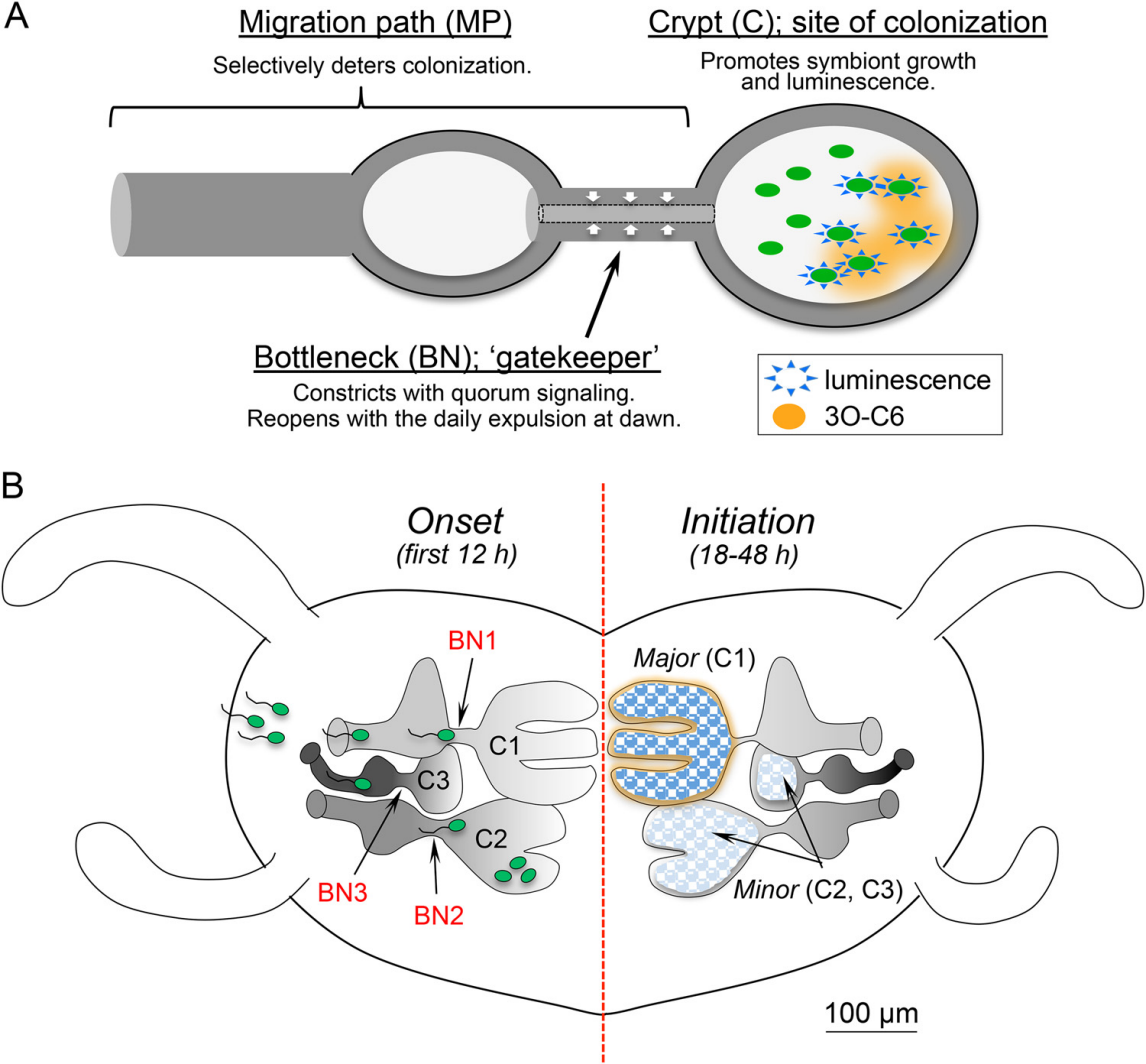
Conceptual model for the symbiont-mediated control of the light organ bottleneck. (A) Colonized crypts contain a symbiont population resulting from the growth of founder cells. As the proliferating symbionts reach a sufficiently high population density, they initiate LuxI-mediated secretion of the quorum-sensing (QS) molecule 3O-C6 (orange), which binds to the receptor LuxR in nearby cells to induce a positive-feedback loop, even in adjacent *luxI* mutant symbionts (93). One result is light production (blue), but other genes are also regulated by LuxIR signaling (11). One or more of those genes trigger a change in actin polymerization within the adjacent bottleneck tissue, causing it to constrict (white arrows). This constriction effectively closes the bottleneck, retaining essentially all symbionts within the crypt, while preventing secondary colonization by other bacteria that may be in the migration path. (B) A light organ contains three pairs of the region simplified in panel A; each pair is at a different stage of development at the onset of symbiosis. V. fischeri cells (green) colonize all three crypt stages. Because of their greater size (39) and ability to support a symbiont population (35), the major crypt pair (C1) may provide a higher concentration of diffusible 3O-C6 (orange) and exert influence on the initiation of symbiosis in the minor crypts, which have less dense populations. QS molecules diffusing from crypts colonized by WT symbionts can exert some influence bottleneck (BN) constriction in an adjacent crypt that lacks symbionts with a functional QS system (i.e., are either empty or contain a *luxI* mutant).

We also asked whether, in CD-treated animals, symbionts were more likely to exit the crypts through the pharmacologically opened bottleneck. Using the intensity of the fluorescence signal of green fluorescent protein (GFP)-expressing WT V. fischeri as a proxy for their abundance, we examined the relative position of symbionts in two regions, the crypt (i.e., in position) or the migration path (i.e., out of position), as a function of the associated bottleneck’s dimension (Fig. 6). As predicted, a nonconstricted bottleneck poorly retained the symbionts in the crypt (Fig. 6A and B’). The increased abundance of V. fischeri cells present in the migration path tissues was significantly correlated with an increase in the bottleneck diameter [*r*(26) = 0.806, *P* < 0.0001], but not with the decreased level of symbionts within the crypts. This effect was at least partially reversible: subsequent dilution of the CD by replacing the seawater around the animals resulted in a narrowing of the bottlenecks, although on average, they did not return to a level of constriction characteristic of untreated tissue (Fig. 6B). Surprisingly, while the bottleneck diameter of Apo light organs was unaffected by CD treatment, about half of the symbiotic bottlenecks became even more open after CD treatment (Fig. 7B) than those of Apo animals. This result suggested that the state of actin polymerization may become more responsive to external conditions after colonization.

### QS-mediated bottleneck constriction is needed to retain symbionts in their crypts.

Because opening a bottleneck by CD treatment resulted in the loss of symbionts from the crypts (Fig. 6B’), we hypothesized that a population of QS mutants would be similarly susceptible to release from their crypt. Typically, after venting of WT cells, only a few symbionts remain stranded in the migration path once the bottleneck has reclosed (35). As a result, the total fluorescence produced by GFP-labeled symbionts in the migration path is typically <10% that detected within the crypts (Fig. 6B’). Because the bottleneck is not fully closed by the *luxI* mutant, the fluorescence intensity (i.e., the number of symbionts) in the migration path was greater than it was for WT, while the fluorescence in the crypts was lower (Fig. 6B’’). Similar to the CD-treated, WT-colonized animals, the level of *luxI* mutants present in the migration path was significantly correlated with bottleneck diameter [*r*(26) = 0.45, *P* < 0.05]. Complementation of *luxI-*colonized light organs with 3O-C6 resulted in a partial recovery of gatekeeper function and a return to a WT-like pattern of no significant correlation between the bottleneck diameter and abundance of symbionts in the migration path [for WT, *r*(26) = 0.31, *P* = 0.051; for the *luxI* mutant plus 3O-C6, *r*(17) = 0.42, *P* = 0.067] (data not shown). This result indicated that a nonconstricted bottleneck was unable to confine the QS-deficient symbionts in its crypt.

We next asked whether, over the first 2 days of symbiosis, proper closing of the bottleneck facilitates selection of particular strains during competition. We hypothesized that the signaling mutant (Δ*luxIR lacZp*-*luxCDABEG*) was selected against in crypt 1 (Fig. 5C) because of its failure to elicit proper bottleneck closure. While the mean closure of the bottleneck of crypt 1 (BN1) containing the dark mutant (Δ*luxCDABEG*) was comparable to the response to the signaling mutant, the range of the responses induced by the dark mutant was greater and even included WT-like levels of closure at the low end (Fig. 5C’). In contrast, the signaling mutant induced a smaller range of wide bottlenecks responses, extending between Apo and wider levels at 24 h. In contrast, the responses of BN2 and BN3 to either the dark or signaling mutant were similar to one another, ranging from WT-like levels of closure to levels only occasionally wider than Apo (Fig. 5C’). These data suggest that not only is the most mature tissue (BN1) more responsive to the activities of strains colonizing its associated crypt but also its response to the signaling mutant encompasses the Apo-like phenotype, a condition that is not conducive to constraining the symbionts in crypt 1. Thus, the increased competitive defect of the signaling mutant (Fig. S6A) correlated with its reduced ability to induce an effective closure of the bottleneck when colonizing crypt 1 (Fig. 5C’).

## DISCUSSION

Persistent associations between microorganisms and their animal hosts are nearly ubiquitous in nature (1, 50, 51), often occurring on the apical surfaces of polarized epithelial tissue. Activities of the bacterial community lining these surfaces can have profound effects not only on the immune system but also on host development. For instance, colonization of neonatal humans by the natural microbiota triggers early developmental processes that are associated with healthy outcomes (21, 26, 52, 53). Here, we examined these processes in the squid-vibrio symbiosis, an experimentally tractable model of beneficial bacterial tissue colonization. Within the developing light organ of the newly hatched host, bottlenecks leading to symbiont-containing crypts constrict after the first bacteria have passed through and proliferated (34, 35). We hypothesized that one purpose of this constriction is to retain the majority of symbionts within the crypts until the daily dawn expulsion (54). Confining symbionts to their site of colonization by closing the entry pathway is not a unique host strategy, having been observed in other beneficial associations like those in the bean bug (55, 56) and the stinkbug Plautia stali (57); however, the role of the symbionts in modulating the closure has remained difficult to examine. Using the squid-vibrio association, we (i) found that this host response requires the LuxIR quorum-sensing (QS) network of the symbionts and (ii) determined the broader biological consequences of the constriction to the association.

### Symbionts trigger bottleneck closure through LuxIR quorum sensing.

We hypothesized that constriction of the bottleneck results from a change in the conformation of actin within the terminal web lining the polarized epithelium that forms the bottleneck (Fig. 6A). Previous work showed that after the crypts are colonized, the duct portion of the migration path (Fig. 1A) undergoes a two- to threefold constriction, a process that was accompanied by an increase in actin abundance but no change in actin transcription (58). This study postulated that the symbionts alter the degree of actin polymerization in the duct tissues via the Arp2/3 actin nucleation complex, not unlike what has been described in the modulation of actin polymerization by pathogenic bacteria (59, 60). Here, we showed that, further along the migration path, symbiont-induced constriction of the bottleneck could be prevented by treatment with either of two inhibitors of actin polymerization. Interestingly, these inhibitors did not increase the diameter of aposymbiotic bottlenecks, suggesting that this “open” conformation is not maintained by actin polymerization.

The observation that bottleneck closure requires the presence of metabolically active V. fischeri within the crypts (35) suggested that this host tissue responds to a symbiont-produced biochemical cue. One common mechanism of bacterial communication is acyl homoserine lactone (AHL) QS, during which many host-associated Gram-negative bacteria secrete extracellular signals to coordinate their activities (e.g., reference 61). In V. fischeri, QS is a sequential process involving two AHL signal synthases, AinS and LuxI, which produce AHLs that induce a regulon of genes required to properly initiate and persist as light organ symbionts (17). Mutant strains with mutations in either of these AHL synthases are as motile and initiate colonization of the light organ as well as the WT does (17), indicating that their effects on host phenotypes occur downstream of accessing the crypts. Colonization by V. fischeri mutants with a defect in one of these two synthases revealed that bottleneck closure is primarily dependent on the late-phase signaling system, in which the LuxI-produced AHL 3O-C6 binds to LuxR (Fig. 3A). However, because C8 also binds LuxR, albeit poorly (62) (Fig. 3A), even in the absence of 3O-C6, some induction of the LuxR regulon, and thus bottleneck closure, was to be expected (Fig. 4).

QS molecules also serve as agents of interkingdom signaling by initiating host responses either directly (61, 63, 64) or indirectly (19). In some associations, such as those of jellyfish (65) and hydra (3), the host can modify these symbiont-generated signals, thereby reciprocally controlling the behavior of the bacteria within their tissues. We do not suspect that this is occurring in the squid host tissues, due to abundant evidence of high levels of LuxI QS when V. fischeri bacteria are associated with the host (14), including LuxI-influenced genes and their products (37, 66). However, 3O-C6 itself does not appear to independently signal host bottleneck closure because its pharmacological addition did not directly induce the characteristic constriction when the symbionts were not present, i.e., when the light organ was either aposymbiotic or cured of symbionts prior to exposure (see Fig. S5B in the supplemental material) or when either strain lacking a functional LuxR (*luxR* or Δ*luxIR lacZp*-*luxCDABEG* mutant) occupied the crypts (Fig. S4A). Further, 3O-C6 delivery by a strain that constitutively expressed the LuxI synthase, while lacking the LuxR receptor (BDB231; Table 1), was unable to induce the constricted-bottleneck phenotype (Fig. S3 and S4A). Instead, the closure appears to be influenced by 3O-C6’s activation of LuxR (Fig. 3C and S4A) and the subsequent downstream effects on the LuxR regulon, a set of 30 genes that includes the luminescence-encoding *luxCDABEG* operon as well as genes encoding a number of efflux proteins, proteases, and other products (11, 67).

Previous studies have reported that bioluminescence was required for most of the differential gene expression in the symbiotic light organ and even some remote tissues like the eyes (19), while colonization by a luminescence mutant caused defects in several host tissue phenotypes (4, 18). In contrast to these reports, bioluminescence was not the salient product of 3O-C6 QS that led to bottleneck closure (Fig. 4), suggesting that one or more of the other two dozen non-*lux* genes in the LuxIR regulon are likely to encode the effector(s) of this host response.

Both pathogens (68–70) and mutualistic symbionts (71–73) secrete proteases and other effectors that target host actin. For example, certain strains of enteropathogenic Escherichia coli (EPEC) that hijack the regulation of host actin within the intestine use a type 3 secretion system to inject effectors that target Arp2/3 complex signaling (74–76). However, unlike EPEC, the population of V. fischeri, at the time of bottleneck constriction, is contained within the crypts and not in contact with the region of host tissue whose actin is modified. This difference suggests that symbionts can modulate actin polymerization while remaining extracellular and several cell layers away from the target tissues (Fig. 7A). Our future work will focus on the secreted proteases and other effectors in the LuxIR regulon as candidates that may modulate actin in the bottleneck, as these have been shown to be induced by 3O-C6 *in vitro* (11, 67).

### Biological implications of bottleneck closure to the symbiosis.

Not surprisingly, the constriction of a stretch of the migration path connecting the external environment with the symbiont-containing crypts impacts both the ecology and behavior of the symbiosis. The closure restricts any secondary colonization after the entry and growth of the initial symbiont (47) and decreases the probability that the crypt population will contain more than one strain (77, 78). Over the long term, promoting a clonal population in each crypt is likely to diminish competition between symbionts (i.e., reference 79) and, ultimately, avoid fitness costs to the host (80). Bottleneck closure is also a key factor in ensuring that the bacterial population is retained within the crypts, where their bioluminescence is critical to the host in its nocturnal behavior (13). Thus, a properly regulated bottleneck has several important roles in the symbiosis.

The developmental state of the host tissues also affects how the bottleneck responds to the symbionts. The light organ of a newly hatched juvenile has three pairs of crypts, with each pair having reached a different level of maturation at the time of hatching (Fig. 1A). The more mature the crypt, the greater its capacity to contain and support symbionts (34, 35, 81). The V. fischeri populations colonizing the less mature and smaller crypts C2 and C3 were less viable (35) and slower to initiate luminescence (40). In addition, the bottleneck associated with the most mature crypt, C1, was more sensitive and exhibited a wider range of responses to the signaling and bioluminescent activity of its symbionts. Given that bottleneck closure underlies symbiont retention, the responsiveness of this gatekeeper may lead to retention of strains with higher levels of QS and light production, and ultimately to different patterns of colonization by crypt type or even possible symbiotic strategies (35). Such a conclusion may also apply to other horizontally acquired symbioses, like those in mammalian microbiomes, where the maturation state of the tissue and its resultant interaction with bacterial symbionts have consequences for immune system development and function (24, 26, 82).

The extent of such biogeographic complexity within a symbiotic organ is a subject of emerging interest for many associations (79, 83–85). Two questions common to all of these systems are as follows. (i) How distinct are the responses of different regions that house beneficial bacteria (i.e., biogeography)? (ii) How might symbionts in these separated regions communicate among themselves? In the case of tissue distinctions, the bottlenecks associated with the major crypts (BN1) displayed different degrees of closure when the associated crypt (C1) was colonized by different strains (Fig. 5C and Fig. S6A’). The more normal level of gatekeeper function by some BN1s might not only account for the less dramatic defect in C1 colonization by the dark mutant compared to the signaling mutant (Fig. 5C, left) but also partially explain the signaling mutant’s defect when competing directly with the dark mutant (Fig. 5C, right).

The evidence presented here leads to two conclusions. (i) The major driver of a bottleneck’s constriction originates from LuxIR activity in the adjacent crypt. (ii) The activity of symbionts in neighboring crypts can, over time, exert a limited effect on other bottlenecks. While we as yet do not know its chemical nature, it seems likely that the LuxIR-induced effector is a poorly diffusible molecule, perhaps a protein. The next step will be to identify this effector through systematic deletion of the other >20 members of the LuxIR regulon and analysis of their ability to induce bottleneck closure.

While bioluminescence has long been recognized as the major currency that symbiotic V. fischeri supply their host (13), we show here that other QS-induced symbiont factors control the key gatekeeper activity of the light organ bottlenecks. Further work is necessary to also define the linkage between the LuxIR regulon and a competitive advantage in the host. A better understanding of the impact of bacterial communication signals on this tissue microenvironment will further elucidate how bacteria can trigger or evade a host response to their presence as they work to sustain a beneficial, or pathogenic, association.

## MATERIALS AND METHODS

### Bacterial strains.

Bacterial strains and plasmids used in this study are summarized in Tables 1 and 3. Vibrio fischeri was grown in Luria-Bertani salt (LBS) (86) with antibiotics where applicable (see Text S1 in the supplemental material), or in seawater tryptone (SWT) medium (87). Subcultures were grown until cells reached mid-log phase of growth prior to their dilution in seawater to inoculate juvenile squid hatchlings.

### Plasmid and mutant construction.

Primers used to create V. fischeri expression and gene deletion plasmids are listed in Table 4. Genomic insertion of *ainS* or *luxI* under the control of their respective endogenous promoters at the genomic *att*Tn*7* site was performed as previously described using a mini-Tn*7* vector (88) (Text S1).

**TABLE 4:** Plasmids used in this study

Primer	Sequence	Restriction site
Primers to insert *luxI* and *ainS* into pEVS107		
luxIF	CTAGCCTAGGCCAATTTGGAGGTTTGGTG	AvrII
luxIR	GTACACTAGTGTTCGAGTATTAATTTGATACAGC	SpeI
ainSF	CTAGCCTAGGCTGAGAAAGTAATTTCCTCAGC	AvrII
ainSR	CTAGACTAGTTTAAACTTTAGGTAAAGTAGTTAAC	SpeI
Primers to insert *luxI* into pVSV105		
luxIF-XbaI	CTAGtctagaGGTTGCATGGCTGTAATG	XbaI
luxIR-KpnI	CTAGggtaccGTTCGAGTATTAATTTGATACAGC	KpnI
Primers to delete *luxIR*		
luxRUSF	GTACggatccGTGAATCAAGTTCGAGTAAATTG	BamHI
luxRUSR	GTACgaattcCGCCATTAATTGTCCATACC	EcoRI
luxIDSF	GTACggtaccCTCGAACATAATACATATAGTTAG	KpnI
luxIDSR	GTACgagctcGTCTTGAGTTGAGAAGCAG	SacI
Primers to insert P_A1/O4/O3_ into *luxIR* deletion construct		
PlacF	GTACgaattcCGATGGTGTCAACGTAAATG	EcoRI
PlacR	GTACggtaccCTGTGTGAAATTGTTATCCGC	KpnI
Primers to insert *luxI* and *ainS* into pEVS107		
luxIF	CTAGCCTAGGCCAATTTGGAGGTTTGGTG	AvrII
luxIR	GTACACTAGTGTTCGAGTATTAATTTGATACAGC	SpeI
ainSF	CTAGCCTAGGCTGAGAAAGTAATTTCCTCAGC	AvrII
ainSR	CTAGACTAGTTTAAACTTTAGGTAAAGTAGTTAAC	SpeI
Primers to insert *luxI* into pVSV105		
luxIF-XbaI	CTAGtctagaGGTTGCATGGCTGTAATG	XbaI
luxIR-KpnI	CTAGggtaccGTTCGAGTATTAATTTGATACAGC	KpnI
Primers to delete *luxIR*		
luxRUSF	GTACggatccGTGAATCAAGTTCGAGTAAATTG	BamHI
luxRUSR	GTACgaattcCGCCATTAATTGTCCATACC	EcoRI
luxIDSF	GTACggtaccCTCGAACATAATACATATAGTTAG	KpnI
luxIDSR	GTACgagctcGTCTTGAGTTGAGAAGCAG	SacI
Primers to insert P_A1/O4/O3_ into *luxIR* deletion construct		
PlacF	GTACgaattcCGATGGTGTCAACGTAAATG	EcoRI
PlacR	GTACggtaccCTGTGTGAAATTGTTATCCGC	KpnI

### Growth curves and luminescence of strains in culture.

Cells were grown in LBS medium to an optical density at 600 nm (OD_600_) of 0.3 and diluted to 0.02 OD with either fresh LBS or SWT. Growth of 1-ml cultures in 24-well clear plates was monitored using a GENios Pro plate reader (Tecan, Morrisville, NC) with continuous shaking at 28°C, and measurements taken every 20 min for 15 h. The specific luminescence of V. fischeri was determined by taking luminometer (TD-20/20; Turner Designs, Inc., Sunnyvale, CA) readings from cultures at an OD of 1.0. Decanal was added to eliminate aldehyde limitation and produce maximum luminescence (87) (see Fig. S5D in the supplemental material).

### Squid colonization assays.

Within 1 to 3 h of hatching, individual E. scolopes juveniles were inoculated in the dark with ∼5,000 cells per ml of V. fischeri cells in seawater, and luminescence was measured to assess bacterial light output after colonization of the animal (14), prior to sacrificing the animal for the assay. To reduce the variation contributing to the phenotypes measured, each experiment included hatchling squid from a single clutch. Symbiont population levels in colonized animals were estimated by plating homogenates of frozen animals and counting CFU arising on LBS medium, as described previously (89).

### Colonization competition between strains carrying fluorescent labels on plasmids.

We found no growth effect of carrying the fluorescent-protein-encoding plasmids (Text S1). To ensure that the competition defect of Δ*luxIR lacZp-lux* in colonization experiments was not due to interactions between strains in culture, we carried out the competitions (described in the legend to Fig. 5) *in vitro* as well. The slight competitive disadvantage of the LuxIR signaling strain in coculture with WT could not account for the 17-fold disadvantage when the strains were competed in colonization assays (Fig. 5B).

### Pharmacological treatments.

C8 and 3O-C6 autoinducers were incubated at 5 μM in seawater with the juvenile squid for 3 h prior to the endpoint of the colonization as previously described (18). Juvenile squid exposed to the ethyl acetate solvent alone in seawater showed no bottleneck response (data not shown).

To remove CD, animals were rinsed with three exchanges of filtered seawater and remained in untreated water for 3 h prior to fixing (see Text S1 for more details).

### Sample fixation and microscopy.

Juveniles were fixed in 4% paraformaldehyde in marine phosphate-buffered saline (mPBS; 0.45 M NaCl in a 50 mM sodium phosphate buffer [pH 7.4]), rinsed, dissected, stained, and mounted as previously described (35; see Text S1). Confocal microscopy was performed using a Zeiss 710 and a Leica SP8 X confocal microscope as previously described (35; see Text S1). For image analysis using FIJI (ImageJ) (90), see Text S1.

### Statistical analysis.

Data were analyzed using GraphPad Prism software, version 7.0 (GraphPad Software, Inc., La Jolla, CA) as previously described (35; see Text S1).

### Mathematical modeling.

Analyses using mixed models were done in R (91) as described previously (92) (see Text S1).
