# Supplementary material for: Bacterial Quorum-Sensing Regulation Induces Morphological Change in a Key Host Tissue during the Euprymna scolopes-Vibrio fischeri Symbiosis
Source: mBio. 2021 Sep 28;12(5):e02402-21. doi: 10.1128/mBio.02402-21 (PMC8546586; doi:10.1128/mBio.02402-21)
Supplement: TEXT S1 [file mbio.02402-21-s0001.pdf]

Bacterial quorum-sensing regulation induces morphological change in a key host tissue during the *Euprymna scolopes*–*Vibrio fischeri* symbiosis

Essock-Burns, T., Bennett, B. D., Arencibia, D., Moriano-Gutierrez, S., Medeiros, M., McFall-Ngai, M. J., and E. G. Ruby
Pacific Biosciences Research Center, University of Hawaiʻi at Mānoa, Honolulu, Hawaiʻi, United States of America

Supplemental Material

MATERIALS AND METHODS

General. All chemicals were purchased from Sigma-Aldrich (St. Louis, MO) and fluorescent dyes from Thermo Fisher Scientific (Waltham, MA), unless otherwise stated. Adult *E. scolopes* animals were collected from Maunalua Bay, Oahu, and maintained as previously described (1). Clutches of eggs and juveniles were incubated in natural offshore seawater on a 12:12 h light-dark (LD) photic cycle.

Bacterial strains. Bacterial strains and plasmids used in this study are summarized in Table 1. *Vibrio fischeri* was grown in Luria-Bertani salt (LBS) medium (10 g of Bacto-Tryptone, 5 g of yeast extract, 20 g of NaCl, and 50 ml of 1 M Tris-HCl buffer [pH 7.5] per liter of deionized water) (2) Overnight cultures were inoculated with single colonies from freshly streaked −80°C stocks and liquid cultures shaken at 225 rpm, 28°C. Antibiotics were added to overnight cultures, where applicable, at the following concentrations: kanamycin (Km), 50 µg/ml; chloramphenicol (Cm), 5 µg/ml; erythromycin (Em), 5 µg/ml. Or overnight cultures were diluted 1:1000 in seawater tryptone (SWT) medium (5 g of Bacto-Tryptone, 3 g of yeast extract, 3 ml of glycerol, 700 ml of filtered seawater and 300 ml of deionized water) (3). Subcultures were grown

until cells reached mid-log phase of growth prior to their dilution in seawater to inoculate juvenile squid hatchlings.

Plasmid and mutant construction. Primers used to create *V. fischeri* expression and gene deletion plasmids are listed in Table 1. Genomic insertion of *ainS* or *luxI* under control of their respective endogenous promoters at the genomic *att*Tn7 site was performed as previously described using a mini-Tn*7* vector (4). Briefly, *ainSp-ainS* or *luxIp-luxI* were cloned from the *V. fischeri* genome and inserted into pEVS107 via *Avr*II and *Spe*I sites. The transposition helper plasmid pUX-BF13 (5) was used to insert the pEVS107 construct at *att*Tn*7*. The *luxI* expression vector was created by cloning *luxI* from the *V. fischeri* genome, and inserting it into pVSV105 (6) via *Xba*I and *Kpn*I sites. Deletion of *luxIR* and replacement with the modified *lac* promoter $P_{A1/O4/O3}$ upstream of *luxCDABEG* was performed similarly, as previously described (7). Briefly, ~1-kb fragments surrounding *luxIR* were cloned from the *V. fischeri* genome and fused via *Eco*RI and *Kpn*I sites to either end of $P_{A1/O4/O3}$ cloned from pAKD601 (8). The fused fragment was inserted via *Bam*HI and *SacI* sites into pSMV3 (9), which carries kanamycin-resistance and *sacB* cassettes. Counter-selection to remove pSMV3 from the *V. fischeri* genome and replace *luxIR* with $P_{A1/O4/O3}$ was performed on high-sucrose, low-salt LB plates (7) at room temperature.

Colonization competition between strains carrying fluorescent labels on plasmids. To determine whether carriage of either the plasmid backbone of *gfp*-carrying pVSV102 or the fluorescent markers CFP and YFP conferred a competitive disadvantage on *V. fischeri* strains during colonization, we competed the WT strain expressing each of the labels against each other, as well as the dark Δ*lux* strain (Δ*luxCDABEG*) carrying the markers, and found no effect of the carriage of the plasmid with either label. Competition experiments were performed by exposing juveniles to two strains with a ~1:1 inoculum (unless otherwise stated), using at least 10 animals per treatment, and were repeated three times. Fluorescently labeled strains were

identified in the plating assays by counting colonies under light passed through filter sets that revealed CFP or YFP using a dissecting microscope (NightSea LLC, Lexington, MA). To ensure that the competition defect of Δ*luxIR lacZp-lux* in colonization experiments was not due to interactions between strains in culture, we carried out the competitions (described in Fig. 5) *in vitro* as well.

Pharmacological treatments. Prior to sample fixation for imaging of the bottleneck portion of light-organ tissue, different chemical agents were incubated in the seawater for 3 h before the endpoint of the juvenile colonization. Two types of autoinducer molecules were used: *N*-octanoyl-L-homoserine lactone (C8-HSL) and *N*-(3-oxohexanoyl)-homoserine lactone (3O-C6-HSL). These compounds were dissolved in ethyl acetate acidified with HCl to produce 5-mM stocks, and kept at −20 °C. Autoinducers were incubated at 5 μM in seawater with the juvenile squid for 3 h prior to the endpoint of the colonization (10). Juvenile squid exposed to ethyl acetate alone in seawater showed no bottleneck response (data not shown).

Two inhibitors of actin polymerization were used. Solutions of cytochalasin D (CD) from *Zygosporium mansoni* (cat no. C8273) in dimethyl sulfoxide (DMSO) were made as a 2 mM stock, and used at several working concentrations to determine bottleneck re-opening (Fig. S9). The CD concentration that relaxed constriction to a level similar to that of an aposymbiotic bottleneck (*i.e.*, 400 nM) was used in subsequent assays (Figs. 6, S9). For the removal of CD and DMSO, animals were rinsed with three exchanges of filtered seawater and remained in untreated water for three hours prior to fixing. The 2-fluoro-*N*-[2-(2-methyl-1H-indol-3-yl) ethyl]-benzamide (CK666) (cat no. SML006) solution was made as an 8.5 mM stock in DMSO, and tested at two concentrations: 20 μM ('high') and 5 μM ('low') (Fig. 7B). The solvent control for these experiments was DMSO at 0.1% (vol/vol).

Sample fixation and microscopy. At the endpoint of colonization assays, juveniles were dropped into 4% paraformaldehyde in marine phosphate-buffered saline (mPBS; 0.45 M NaCl in a 50 mM sodium phosphate buffer, pH 7.4) and fixed overnight at 4 °C with rotation. Following three washes with mPBS, light organs were dissected and permeabilized and stained in 0.1% Triton-X 100 stained with rhodamine phalloidin for F-actin and TO-PRO-3 to label nuclei for two days at 4 °C with rotation. Briefly, washed samples were mounted and imaged on a Zeiss LSM 710 (Carl Zeiss AG, Jena, Germany) confocal microscope as well as a Leica SP8 X confocal microscope (Leica Camera AG, Wetzlar, Germany) as described previously (11). Each side of a light organ (crypts 1-3) was considered one set of measurements. For image analysis, Fiji (ImageJ) (12) was used to generate Z-projections of sub-stacks to generate an unobstructed view of the bottleneck portion of tissue, using the phalloidin channel. In the software, a straight line was drawn between the F-actin rich, terminal web at the narrowest point of the bottleneck structure and used as the estimation of the diameter. While the average wild-type bottleneck diameter at early stages of symbiotic development was reduced to 3 µm or less (Fig. 2B), we used a conservative cutoff of >5 µm to operationally define any response to a mutant as different from wild type when comparing across experiments; *i.e.*, the mean response to wild type was always a constriction to less than 5 µm (Table 2).

For intensity measurements to estimate abundance of *V. fischeri* labeled with green fluorescent protein (GFP) in specific tissue regions, all light organs were imaged with the same laser intensity and gain on the detector, and done on the same day. In Fiji, projections of the GFP channel were used with the freeform tool to draw the regions of migration path (MP) 1 and crypt (C) 1 (regions shown in Fig. 7A). The final intensity measurement used was the mean intensity per unit area of each specified region with the background (the fluorescence of tissue away from the two regions of interest) subtracted.

Statistical analysis. Data were analyzed using GraphPad Prism software, version 7.0 (GraphPad Software, Inc., La Jolla, CA), and were first tested for normality using a D'Agostino-Pearson (omnibus K2) test. When data passed this criterion ($P > 0.05$), parametric tests including a one-way ANOVA or two-way ANOVA with time as a factor were used to examine differences between the groups. When the difference was considered significant, then a Tukey or Holm's post-hoc test with a one-way ANOVA or a Sidak's multiple comparison's test for a two-way ANOVA was performed. If data did not pass the normality test, a Kruskal-Wallis test and Dunn's post-hoc test were used to determine differences between groups. Significance indicated by asterisks as follows: * $P < 0.05$, ** $P < 0.01$, *** $P < 0.001$, **** $P < 0.0001$.

Mathematical modeling. Data were analyzed using the program R (13) and the package for linear mixed models (lme4) (14) as described previously (15). Three sets of data were analyzed using mathematical modeling approaches. First, to assess bottleneck responses to strains and compare across experiments (see data in Figs. 2B, S2) we used a mixed model with a random effect structure for host individual nested in experiment. Strain type and time were added to the model as fixed effects. Comparison of the nested model showed a significant interaction effect of time and strain type on the bottleneck responses. Two nested models, one with the interaction effect and one without, were compared with a log likelihood ratio test.

To analyze the data from the co-inoculation with WT, Δ*luxCDABEG* (dark mutant), and Δ*luxIR lacZp-lux* (signaling but luminous mutant), the strains were scored in terms of their LuxI-LuxR functionality (Table S1; see data in Figs. 5, S4). Strains with LuxIR functionality included WT and Δ*luxCDABEG*, whereas Δ*luxIR lacZp-lux* was excluded from this group. We used a nearest neighbor analysis to determine if there was an effect of LuxIR functional strains in nearby crypts to a given focal crypt. We treated an adjacent crypt on the same side of the light organ and the opposing side's crypt 1 when evaluating a focal crypt 1 due to the proximity of the

blind ends of each C1. Data were log-transformed and a linear mixed model fit by maximum likelihood was used to compare across all combinations of focal crypt function for neighborhood effect.

A third analysis tested differences in the luminescence production (light functionality) of strains to assess if the restoration of light in the Δ*luxIR lacZp-luxCDABEG* strain had the same impact on the bottleneck as did wild-type light (Table S1). Empty or Δ*luxCDABEG*-colonized crypts were scored as non-luminous, whereas wild-type light and the restored light of Δ*luxIR lacZp-luxCDABEG* were categorized separately. We used a mixed model to compare whether treating light from both strains as the same (only two classifications, absent or present) was a better characterization or if the model fit better when light was split into the three groups (absent, present, or restored). A linear mixed model was fit by maximum likelihood framework. We assessed fit by AIC with a broad distinction where a ΔAIC of 2 indicated a significantly better fit.

REFERENCES

1. Montgomery MK, McFall-Ngai MJ. 1993. Embryonic development of the light organ of the sepiolid squid *Euprymna scolopes* Berry. Biol Bull 184:296–308. https://doi.org/10.2307/1542448.
2. Dunlap P V. 1989. Regulation of luminescence by cyclic AMP in cya-like and crp-like mutants of *Vibrio fischeri*. J Bacteriol 171:1199–1202. https://doi.org/10.1128/jb.171.2.1199-1202.1989.
3. Boettcher KJ, Ruby EG. 1990. Depressed light emission by symbiotic by *Vibrio fischeri* of the sepiolid squid *Euprymma scolopes*. J Bacteriol 172:3701–3706.
4. McCann J, Stabb E V., Millikan DS, Ruby EG. 2003. Population dynamics of *Vibrio fischeri* during infection of *Euprymna scolopes*. Appl Environ Microbiol 69:5928–5934. https://doi.org/10.1128/AEM.69.10.5928-5934.2003.
5. Bao Y, Lies DP, Fu H, Roberts GP. 1991. An improved Tn*7*-based system for the single-copy insertion of cloned genes into chromosomes of gram-negative bacteria. Gene 109.1:167–168. https://doi.org/10.1016/0378-1119(91)90604-A.
6. Dunn AK, Millikan DS, Adin DM, Bose JL, Stabb E V. 2006. New rfp - and pES213-derived tools for analyzing symbiotic *Vibrio fischeri* reveal patterns of infection and *lux* expression in situ. Appl Environ Microbiol 72:802–810. https://doi.org/10.1128/AEM.72.1.802.
7. Bennett B, Essock-Burns T, Ruby EG. 2020. HbtR, a heterofunctional homolog of the virulence regulator TcpP, facilitates the transition between symbiotic and planktonic

lifestyles in *Vibrio fischeri*. mBio 11:2020.03.31.019356. https://doi.org/10.1101/2020.03.31.019356.
8. Dunn AK, Stabb E V. 2008. The twin arginine translocation system contributes to symbiotic colonization of *Euprymna scolopes* by *Vibrio fischeri*. FEMS Microbiol Lett 279:251–258. https://doi.org/10.1111/j.1574-6968.2007.01043.x.
9. Coursolle D, Gralnick JA. 2010. Modularity of the Mtr respiratory pathway of *Shewanella oneidensis* strain MR-1. Mol Microbiol 77:995–1008. https://doi.org/10.1111/j.1365-2958.2010.07266.x.
10. Chun CK, Troll J V., Koroleva I, Brown B, Manzella L, Snir E, Almabrazi H, Scheetz TE, De Fatima Bonaldo M, Casavant TL, Soares MB, Ruby EG, McFall-Ngai MJ. 2008. Effects of colonization, luminescence, and autoinducer on host transcription during development of the squid-vibrio association. Proc Natl Acad Sci U S A 105:11323–11328. https://doi.org/10.1073/pnas.0802369105.
11. Essock-Burns T, Bongrand C, Goldman WE, Ruby EG, McFall-Ngai MJ. 2020. Interactions of symbiotic partners drive the development of a complex biogeography in the squid-vibrio symbiosis. mBio 11:e00853-20. https://doi.org/10.1128/mBio.00853-20.
12. Schindelin J, Arganda-Carreras I, Frise E, Kaynig V, Longair M, Pietzsch T, Preibisch S, Rueden C, Saalfeld S, Schmid B, Tinevez JY, White DJ, Hartenstein V, Eliceiri K, Tomancak P, Cardona A. 2012. Fiji: An open-source platform for biological-image analysis. Nat Methods 9:676–682. https://doi.org/10.1038/nmeth.2019.
13. Team RC. 2020. R: a language and environment for statistical computing. R Found Stat Comput.
14. Bates D, Mächler M, Bolker BM, Walker SC. 2015. Fitting linear mixed-effects models using lme4. J Stat Softw 67. https://doi.org/10.18637/jss.v067.i01.
15. Seabourn P, Spafford H, Yoneishi N, Medeiros M. 2020. The *Aedes albopictus* (Diptera: Culicidae) microbiome varies spatially and with Ascogregarine infection. PLoS Negl Trop Dis 14:1–21. https://doi.org/10.1371/journal.pntd.0008615.
